# Machine and Deep Learning Models for Hypoxemia Severity Triage in CBRNE Emergencies

**DOI:** 10.3390/diagnostics14232763

**Published:** 2024-12-08

**Authors:** Santino Nanini, Mariem Abid, Yassir Mamouni, Arnaud Wiedemann, Philippe Jouvet, Stephane Bourassa

**Affiliations:** 1 Research Center CHU Ste-Justine Centre Hospitalier Universitaire Mère-Enfant, 3175 Boulevard de la Côte-Sainte-Catherine Drive, Montréal, QC H3T 1C5, Canada; arnaud.wiedemann.med@ssss.gouv.qc.ca (A.W.);; 2 Faculty of Medicine, Université de Montréal, 2900 Boulevard Edouard-Montpetit, Montréal, QC H3T 1J4, Canada; 3 Clinical Decision Support System Articificial Intelligence Health Cluster in Acute Child Care, PE-DIATRICS, CHU Ste-Justine Centre Hospitalier Universitaire Mère-Enfant, 3175 Boulevard de la Côte-Sainte-Catherine Drive, Montréal, QC H3T 1C5, Canadamariem.abid@applicare.ai (M.A.); 4 Department of Mechanical Engineering, École de technologie supérieure (ÉTS), Université du Québec, Montréal, QC G1K 9H7, Canada; 5 Solutions Applicare AI Inc., Montreal, QC H7L 4W3, Canada; 6 Faculté des arts et des sciences, Département d’informatique et de recherche opérationnelle (DIRO), Université de Montréal, 3150 Rue Jean-Brillant, Montréal, QC H3T 1N8, Canada; 7 MEDINT CBRNE Group, 1100 René-Lévesque Blvd W 25 étage, Montréal, QC H3B 5C9, Canada; 8 Mila-Institut Québécois d’Intelligence Artificielle, 6666 Rue Saint-Urbain, Montréal, QC H2S 3H1, Canada

**Keywords:** hypoxemia, machine learning, patient triage, disaster management, CBRNE events, VIMY Multi-System, gradient boosting models, NEWS2+, data preprocessing, feature importance, LSTM, GRU, time series interpolation, deep learning, imputation, interpolation, sliding window, masks, early warning scores, EWS, artificial intelligence, XGBoost, CatBoost, LightGBM, random forest, Tree-based models, voting classifier ensemble, MIMIC-III, MIMIC-IV

## Abstract

Background/Objectives: This study develops machine learning (ML) models to predict hypoxemia severity during emergency triage, particularly in Chemical, Biological, Radiological, Nuclear, and Explosive (CBRNE) scenarios, using physiological data from medical-grade sensors. Methods: Tree-based models (TBMs) such as XGBoost, LightGBM, CatBoost, Random Forests (RFs), Voting Classifier ensembles, and sequential models (LSTM, GRU) were trained on the MIMIC-III and IV datasets. A preprocessing pipeline addressed missing data, class imbalances, and synthetic data flagged with masks. Models were evaluated using a 5-min prediction window with minute-level interpolations for timely interventions. Results: TBMs outperformed sequential models in speed, interpretability, and reliability, making them better suited for real-time decision-making. Feature importance analysis identified six key physiological variables from the enhanced NEWS2+ score and emphasized the value of mask and score features for transparency. Voting Classifier ensembles showed slight metric gains but did not outperform individually optimized models, facing a precision-sensitivity tradeoff and slightly lower F1-scores for key severity levels. Conclusions: TBMs were effective for real-time hypoxemia prediction, while sequential models, though better at temporal handling, were computationally costly. This study highlights ML’s potential to improve triage systems and reduce alarm fatigue, with future plans to incorporate multi-hospital datasets for broader applicability.

## 1. Introduction

Rapid and accurate patient triage is essential during disaster situations, especially in Chemical, Biological, Radiological, Nuclear, and Explosive (CBRNE) events [1,2,3,4]. These incidents often lead to mass casualties and chaotic environments, overwhelming traditional triage systems. The VIMY Multi-System is a technologically enhanced response platform designed to improve casualty management in these complex scenarios. Initiated at the Grouping in AI acute care for the child (CHU Ste Justine, Montreal, QC, Canada) (https://www.chusj-sip-ia.ca/) as part of the VIMY research program, this system leverages artificial intelligence (AI) to manage casualties in disaster settings, including CBRNE events.

**Figure 1:**
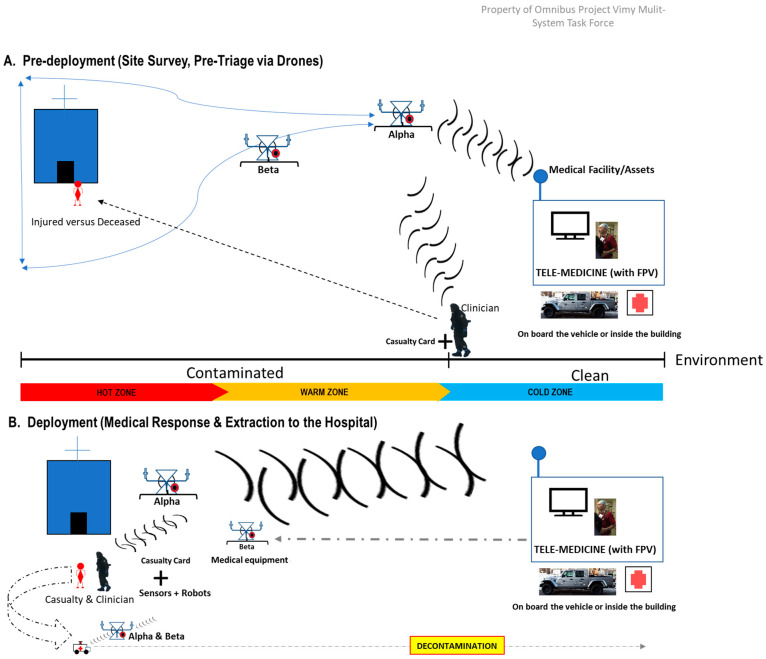
**An illustration that shows the VIMY Multisystem deployment** [8], at a glance, during a medical response in a contaminated environment. Our goal is to develop a proof of concept for AI models that will ensure effective triage in CBRNE environments and other pre-clinical settings.

**Figure 2:**
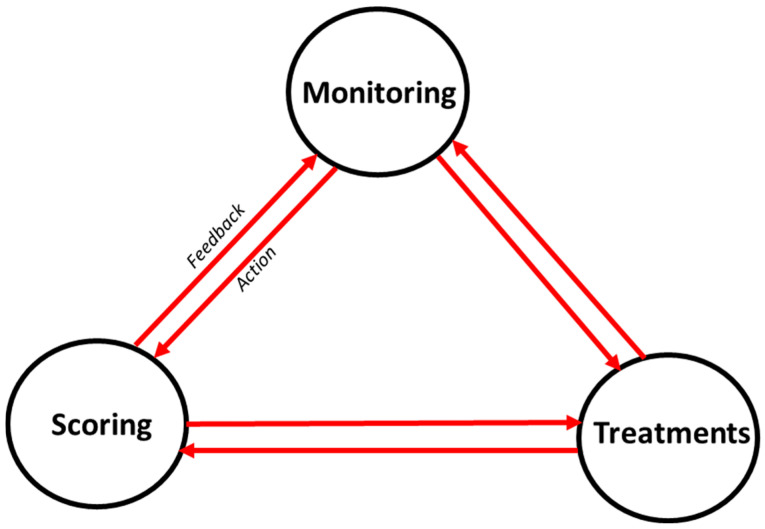
**The illustration represents a simulated hypoxemia condition, testing the monitoring-scoring-treatment nexus through an iterative process** [8]. 1. **Monitoring**: Continuous monitoring of vital signs provides real-time data on key physiological variables such as respiratory rate, temperature, blood pressure, heart rate, and SpO_2_ (oxygen saturation). Both real and simulated data are analyzed to detect correlations and patterns in these digital biomarkers. This analysis helps in predicting the severity score of hypoxemia. 2. **Scoring**: Based on SpO_2_ levels, a hypoxemia severity score is determined, ranging from 0 (best) to 3 (worst). This predicted severity score informs triage decisions, categorizing the patient’s condition as Stat, Urgent, or Stable. 3. **Treatment Administration**: Depending on the severity score, appropriate treatments are administered. For instance, a patient with low oxygen saturation may receive oxygen therapy (with or without an oxygen mask) to stabilize their condition.

More specifically, the VIMY Multi-System [5,6,7,8] is a field-deployable intensive care unit that integrates AI, sensors, and decision-making algorithms to improve healthcare during disasters (Figure 1). The core framework of the VIMY system incorporates an Electronic Casualty Card System (ECCS), functioning as a dashboard that integrates monitoring, scoring, and treatment data (Figure 2). Its primary aim is to equip frontline personnel with advanced tools to overcome the limitations of current triage and early warning systems (EWSs), particularly in CBRNE scenarios, by harnessing machine learning to enhance patient care. This paper specifically focuses on the development of predictive algorithms that are fundamental to the ECCS, thereby contributing directly to the improvement of these critical systems within the VIMY project.

EWSs are tools used in healthcare to assess the severity of a patient’s condition by monitoring key physiological parameters, such as heart rate, blood pressure, respiratory rate, temperature, and level of consciousness. EWSs help healthcare providers identify patients at risk of deterioration, enabling timely interventions. By tracking vital signs and assigning scores based on their values, EWSs aid in the early detection of patients who may need closer monitoring or medical intervention.

EWS such as NEWS2 [9,10,11,12], PWES [13,14,15,16], the Pediatric Vital Signs Normal Ranges from the Iowa Head and Neck Protocols [17,18], and the Modified Early Warning Score (MEWS) [19,20,21] have been developed to monitor patients’ vital signs [11,22,23]. However, existing EWSs do not predict the worsening of a patient’s condition, which would be crucial for early intervention, especially in pre-clinical scenarios like CBRNE events [11,23,24], where multiple patients are injured simultaneously [24,25].

Machine learning (ML) offers a promising approach to enhance the predictive capabilities of EWS by analyzing complex physiological data [26,27]. In this study, we present the development of ML-based models using data from medical devices to predict hypoxemia severity, aiming to improve triage efficiency and reduce medical staff fatigue. Our models leverage physiological and demographic data (including age, which influences the medical interpretation of physiological constants), focusing on key vital signs such as respiratory rate, SpO_2_ (oxygen saturation) levels, heart rate, both systolic and diastolic blood pressure, and temperature. These vital signs parameters are key indicators of patients’ physiological status and are crucial for predicting the severity of hypoxemia, as they directly reflect respiratory and cardiovascular functions. In a preclinical context such as CBRNE scenarios, the goal is to rely on a minimal yet highly informative set of data to support rapid decision-making. These parameters, derived from the NEWS2 score, are easily measurable through wearable connected devices, ensuring their availability and practicality in high-stakes environments. Their integration into automated triage models further emphasizes their clinical relevance, enabling real-time predictions to guide timely and precise interventions. This streamlined approach maximizes efficiency while ensuring critical data are readily accessible for decision-making.

Hypoxemia, characterized by low oxygen levels in the blood, is a common and critical condition in disaster settings. Accurate assessment of hypoxemia severity is essential for timely intervention. Current efforts to use ML to predict hypoxic events are happening in various settings and showcasing diverse methodologies. The studies differ considerably in patient populations, outcome definitions, predictive features, and ML algorithms, making it hard to generalize their conclusions. Consequently, comparing and evaluating these studies comprehensively is quite challenging [28]. Indeed, a systematic review compares [28] past efforts to predict hypoxic events in hospital settings using machine learning, focusing on methodologies, predictive performance, and the populations assessed. The authors identified 12 studies that predicted hypoxic events or hypoxia markers across various settings, including operating rooms, ICUs, and general care units. The machine learning models applied were based on both conventional ML and deep learning methods. Most studies defined their prediction endpoints using specific thresholds for blood oxygen measurements. Clinical variables included patient characteristics, vital signs, and laboratory data, with blood oxygen data (such as peripheral oxygen saturation, SpO_2_) being the most frequently used predictor for hypoxia. However, deep learning and conventional ML methods are not directly comparable, as they were applied to different datasets, and performance metrics were inconsistently reported. Additionally, comparability between studies was hindered by the wide variability in approaches, including the differing settings, which introduced various influences on blood oxygen saturation.

Moreover, a heterogeneous medical population is beneficial for developing a broadly applicable predictive model for hypoxia in CBRNE situations, as it increases the likelihood of achieving generalized results.

Additionally, we believe that the assumption that the model may rely on correlations and patterns among features to build its representations—potentially diverging from the established medical gold standards on which Early Warning Scores (EWS) are based—was not sufficiently developed to be considered a fundamental hypothesis.

In medicine, a “gold standard” refers to the most trusted and conventional method for diagnosing diseases, assessing treatment effectiveness, or verifying the accuracy of tests and measurements. This benchmark serves as the reference standard for comparing alternative approaches.

In this study, we used datasets from MIMIC-III and IV [29,30,31], employing Gradient Boosting Models [32,33,34] (XGBoost, LightGBM, CatBoost), Random Forest (RF), Voting Classifier ensembles, and sequential models [35,36,37] (LSTM, GRU) to predict hypoxemia severity scores. These scores, which actually are the labels we aim to predict, were based on the newly designed NEWS2+ system adapted for pre-clinical scenarios, like CBRNE events. This adaptation, defined by medical expert annotations in the VIMY research group, includes hypoxemia severity for three population groups (adults with COPD, adults without COPD, and pediatric patients without COPD) and a modified EWS for two population groups (adult and pediatric patients). Our comprehensive preprocessing pipeline addressed missing data and class imbalances, ensuring robust and reliable model training.

This paper is structured as follows: Section 2 details the materials and methods, including data preprocessing and model development. Section 3 presents the results of our experiments. In Section 4, we discuss the implications of our findings. Finally, Section 5 concludes the study and outlines future research directions.

## 2. Materials and Methods

### 2.1. Data Sources and Libraries

**Data Sources**

We used the MIMIC-III and MIMIC-IV databases [29,30,31], which are extensive, de-identified health records of ICU patients. MIMIC-IV is an excellent choice for beginners or those seeking to leverage well-established concepts, as it offers a robust and widely used dataset. However, while the HiRID database provides data at 2-min intervals, MIMIC remains the most comprehensive dataset, offering greater breadth and depth for a variety of clinical research applications.

Given that, to our knowledge, no public physiological dataset has been collected specifically in a CBRNE context, we used ICU data for this proof of concept. Critical care patients frequently exhibit severe distress patterns akin to those expected in CBRNE scenarios, making these data a suitable proxy.

Furthermore, we have a very heterogeneous population, which can actually be beneficial, as our goal is to predict hypoxia in CBRNE situations in a non-specific manner. Indeed, rather than targeting a single scenario, we aim to develop a broadly applicable predictive model. Thus, a diverse population increases the likelihood of achieving broadly applicable results, enhancing the overall scope of our predictive model.

Finally, the MIMIC-III dataset includes around 58,000 hospital admissions for over 40,000 unique patients, while MIMIC-IV expands this to over 380,000 admissions for more than 210,000 patients from 2008 to 2019. Both databases offer detailed information, including continuous vital sign monitoring (recorded regularly or very frequently), laboratory results, and demographic details, making them highly applicable to our hypoxemia prediction study.

**Libraries and Tools Utilized**

This project employed a variety of Python libraries for data processing, visualization, and machine learning.

Core operations were supported by libraries such as os, math, time, json, string, random, and warnings. Data manipulation and analysis were carried out using pandas, numpy, and scipy.stats, with additional support from openpyxl, tables, and pickle for file handling and data storage. Visualization was performed using matplotlib.pyplot and seaborn, enabling clear and informative data analysis.

Machine learning tasks were implemented with scikit-learn, which provided tools for model selection, evaluation, and preprocessing. Ensemble methods such as RandomForestClassifier and VotingClassifier were extensively utilized. Gradient boosting frameworks, including xgboost, catboost, and lightgbm, were employed for their high performance and efficiency. Hyperparameter optimization was conducted using hyperopt.

Deep learning, specifically for sequential models, was carried out using PyTorch. Key components included Dataset, DataLoader, and torch.nn for designing and training models. Parallelism and performance optimization were achieved with joblib, concurrent.futures, and gc, while progress tracking and visualization were facilitated using tqdm and torch.utils.tensorboard.

Development was conducted in the Google Colab paid Pro version, which enabled seamless data integration via drive.mount and provide a robust platform for experimentation. This comprehensive library suite supported the efficient and effective implementation of advanced machine learning and sequential modeling techniques.

### 2.2. Inclusion Criteria and Data Representativeness

**Figure 3:**
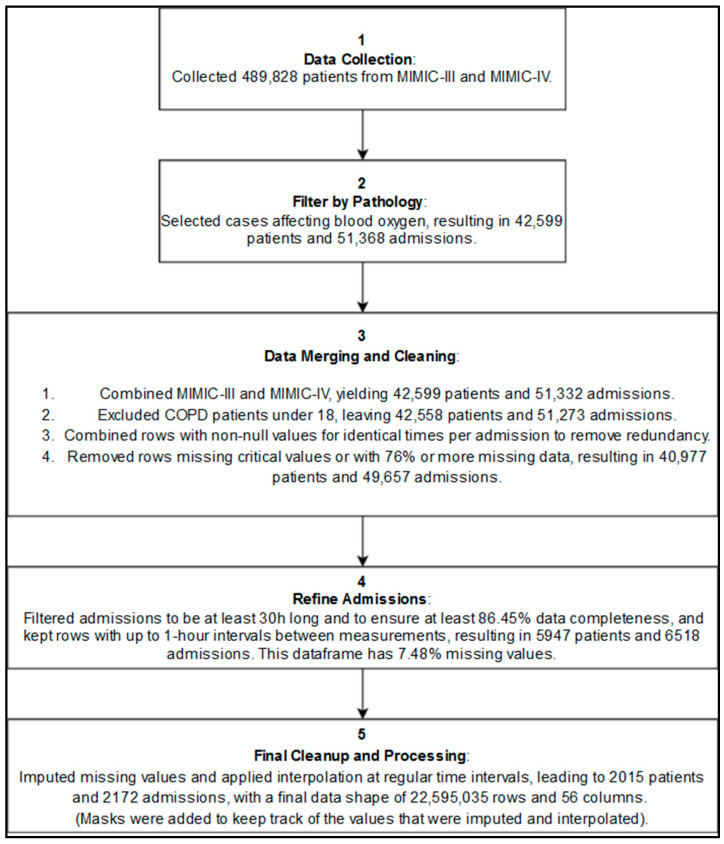
**Simplified inclusion diagram showing the main preprocessing steps before the imputations and interpolations and after.** See the complete diagram in Appendix A.

Patients were included if they had recorded episodes of hypoxemia or were diagnosed with conditions very often associated with potential low blood oxygenation levels, which made them more likely to experience hypoxemic episodes (see Appendix A Figure A6). A total of 51,368 admissions from 42,599 unique patients were selected based on the International Classification of Diseases (ICD) codes related to hypoxemia and respiratory distress. Figure 3 illustrates the simplified inclusion process.

### 2.3. Data Preprocessing

#### 2.3.1. Labeling and Feature Engineering

The VIMY project research team has initiated a new research wing focused on triage and early-warning systems to support the development of the NEWS2+ early-warning system. NEWS2+ is an extension of the original NEWS2, being revisited in light of recent advancements in the scientific literature, and relevant to pre-clinical contexts. Specifically, the medical experts on the team have focused on adapting the original NEWS2 parameters for application in acute pre-hospital environments. We have developed an alternative method to “inform” the model about what constitutes a trigger value for the physiological variables in question, incorporating an alarm mechanism. Additionally, temporal information is provided for each physiological variable of interest, detailing how long each alarm persisted. Medical experts within the VIMY team have contributed significantly to this initiative, and the adapted chart will be extended for use in disaster scenarios, including CBRNE events.

**Figure 4:**
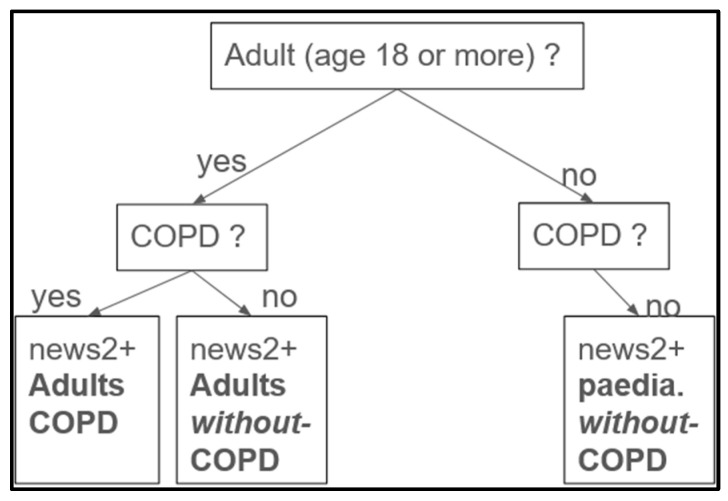
**Labellisation process overview**. Designed on a decision-tree like system. Our process included no children (patients < 18 years) with COPD, only without. For each of the three categories, we used different threshold values for SpO_2_ (%) to determine the severity scores. “paedia”. stands for paediatric.

***Use of SpO_2_ for categorization.*** To categorize hypoxemia severity, we introduced a severity labeling column based on SpO_2_ values, classifying patients as severe, moderate, mild, or normal. SpO_2_ was chosen as the central parameter due to its prevalence and accessibility through oximeters in pre-hospital and acute care scenarios. The system assigns scores ranging from 0 (normal) to 3 (severe) based on SpO_2_ levels, with adjustments for patient age and the presence or absence (Figure 4) of chronic obstructive pulmonary disease (COPD).

In certain situations, gold-standard thresholds need to be adapted based on advancements in the literature. For instance, Dempsey et al. [38] described hypoxemia levels in healthy individuals as follows: mild (93–95%), moderate (88–93%), and severe (<88%). Bourassa et al. [8] proposed hypoxemia thresholds of <90% for the general population and <88% for COPD patients in room air conditions [8]. They also set hypoxemia thresholds for supplemental oxygen therapy at >92% for the general population and >94% for those with COPD.

Additionally, Johannigman et al. [39] reported notable changes in SpO_2_ during aeromedical evacuations, where 90% of military personnel experienced at least one desaturation event with SpO_2_ <90%, and over half dropped below 85%

From the medical conditions manually selected in the MIMIC database, COPD cases were identified based on the presence of the following conditions: “Chronic Obstructive Respiratory Disease”, “COPD arising in the perinatal period”, “Chronic obstructive asthma with status asthmaticus”, and “Other chronic bronchitis”, which could have an impact on the SpO_2_ level in a basal state.

Table 1 displays the adapted values for different hypoxemia levels in adults with and without COPD, as well as in the pediatric population without COPD (pediatric population with COPD is not considered in this study). By providing tailored SpO_2_ thresholds for varying patient needs, the NEWS2+ scoring matrices support comprehensive hypoxemia risk assessment and facilitate targeted clinical responses across diverse patient populations.

***Feature Engineering*** Table 2 introduces NEWS2+, an expanded version of the National Early Warning Score 2 (NEWS2), designed to provide a comprehensive assessment of patient status. Developed with the guidance of the VIMY team’s medical experts, NEWS2+ evaluates vital signs using specific thresholds and severity levels derived from established Gold Standards. The matrices presented outline the NEWS2+ scoring system applied to six primary physiological variables—respiratory rate, oxygen saturation (SpO_2_), heart rate, systolic blood pressure, diastolic blood pressure, and temperature. These scores function as indicators of abnormal vital signs, supporting enhanced data interpretation and predictive modeling.

Each physiological variable is assigned a score that reflects its deviation from age-adjusted reference values, offering critical insights for hypoxemia prediction. These NEWS2+-derived feature scores, occasionally referred to as “TAG” for each physiological variable, may be used interchangeably in this work.

Table 2 below presents the NEWS2+ matrices used to generate these score features (TAGs), demonstrating the process of assigning individual scores to each physiological variable.

#### 2.3.2. Handling Missing Data and Masks

A comprehensive pipeline addressed missing data through imputation and interpolation methods. Missing data accounted for 7.48% of the dataset. We employed a multivariate imputation strategy: Multiple Imputation by Chained Equations (MICE) [41,42,43] using Histogram-based Gradient Boosting.

Synthetic data entries were flagged using masks to ensure transparency, in line with recommendations from previous research [42,43,44,45], which are critical for trust in AI-driven decision-making [46]. This approach helps inform the model about which values were imputed, improving interpretability. Specifically, each feature column had an associated mask column, where a value of 0 indicated no synthetic data and a value of 1 indicated the presence of synthetic data in that row.

To address irregular time intervals between measurements, we used interpolation to generate regular time series data. Minute-level interpolations were performed using linear interpolation, chosen after comparing it with polynomial and cubic spline methods. Linear interpolation produced the most reliable results for vital signs data, minimizing the risk of implausible physiological values (Table 3). For a quick overview of admissions after imputations and minute-level linear interpolations of key physiological variables, refer to Appendix A Figure A7.

We added a mask for the charttimes—the timestamps (at the minute) corresponding to when each set of measurements was recorded—indicating which rows were interpolated to inform the models about synthetic data (same logic as for the imputed values before). This approach aligns with the intuition that linear interpolation can represent gradual changes in a patient’s condition between successive measurements.

#### 2.3.3. Prediction Window

To determine an appropriate time window for prediction, we analyzed the average duration of each hypoxemia severity level after interpolation. The mean and median durations for severity scores 0 to 3 were calculated, with severity score 3 having a mean duration of 114.8 min and a median of 60.0 min. Drawing from these observations, practical considerations, discussions with our team’s medical expert, and findings from previous studies [28,47,48], we established a 5-min prediction window as the standard for our models. This timeframe is long enough to allow doctors sufficient time to intervene, while remaining short enough to capture near-term risks that require immediate attention. Additionally, it minimizes the risk of inaccurately predicting events that are too far in the future. This balance ensures timely and actionable medical interventions.

The sliding window approach for the sequential models is based on this (see Section 2.6.3).

#### 2.3.4. Data Cleaning and Transformation

- **Duplicated Rows and Admissions Removal:** To address potential redundant rows introduced during preprocessing, we merged rows with non-null values recorded at the same charttime (i.e., the same timestamp) within each admission. For eventual duplicate admissions across the combined MIMIC III and IV datasets (which may have different admission IDs), only the most recent occurrence of each redundant row (considering all physiological and demographic features) was retained, while earlier rows were removed to maintain data consistency. This approach ensures that, for each potentially redundant admission, only a single, complete version remains in the final dataset, effectively eliminating duplicate admissions on a row-by-row basis.
- **Outlier Handling:** Implausible physiological measurements were replaced with NaN and subsequently imputed to address potential human errors in the electronic health records. Specifically, for six key vital signs variables, we excluded values outside defined ranges: respiratory rate, heart rate, and both systolic and diastolic blood pressure above 300 or below 0; SpO_2_ above 100 or below 0; and temperature readings above 60 or below 0. To ensure consistency, this step was applied both before and after imputation and interpolation.
- **Data Rounding:** Physiological values were rounded in order to standardize precision, which supports model generalization and may reduce the likelihood of overfitting, especially as future work integrates data from additional hospitals.
- **Missing values**: Imputation by Chained Equations (MICE) using Histogram-based Gradient Boosting was carried out to complete the 7.48% missing values from our preprocessed dataframe
- **Time Alignment:** Interpolated data at minute-level intervals to standardize time steps across admissions (synthetic data).

#### 2.3.5. Data Splitting

To ensure robust comparisons and avoid data leakage, patient data were split into training, validation, and testing sets, ensuring no patient appeared in more than one set (patient wise not admission wise). Specifically, 75% of patients were allocated to the training set (2015 patients), while the validation and test sets each comprised 12.5% of the patients (212 patients each).

### 2.4. Exploratory Data Analysis (EDA)

Refer to Section 2.6.2 for a clear and concise summary of all features, including their descriptions, types (outlined in the legend), and measurement units.

- Additionally, we performed Exploratory Data Analysis (EDA) to examine data distributions and uncover correlations. **Demographics and features:** Table 4 illustrates the patients’ age, gender, race, and ethnicity distributions.
  Concerning the demographics, for the races and ethnicities, we referred to the United States Census Bureau [49].
  The age stratifications shown below were a constitution work from various sources [50,51] and were used to calculate the NEWS2+-derived feature scores (TAGS) for the six key physiological parameters.

Table 5 below details the features after preprocessing used for model training.

**Figure 5:**
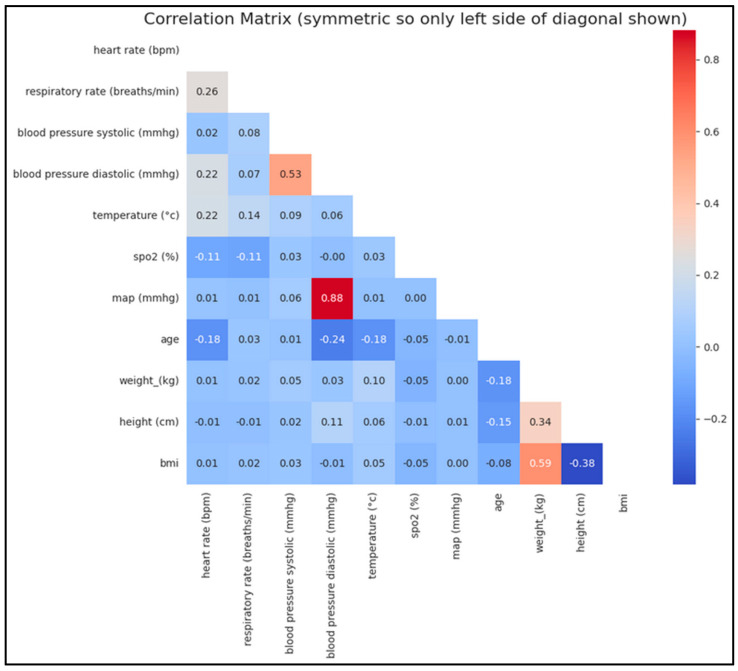
**Correlation matrix of main physiological variables before imputations and interpolations.** There is a strong positive correlation between mean arterial pressure (MAP) and diastolic blood pressure (BP), which is expected since MAP is derived from both diastolic and systolic BP (mathematical relation). Similarly, a strong positive correlation between systolic and diastolic BP aligns with physiological norms. As anticipated, weight and BMI are also strongly correlated, given that BMI is calculated from weight. Conversely, a negative correlation between BMI and height is observed, as BMI decreases with increasing height. Additionally, within a certain age range, a negative correlation between age and diastolic BP can be expected. These correlations underscore the physiological relationships between these variables. Both BMI and MAP are physiologically significant and may provide valuable insights for predicting the risk or severity of hypoxemia.

- **Label Distribution:** We observed an unbalanced dataset, where higher severity scores occur less frequently (Table 6). The labels, defined as 0 (normal), 1 (mild), 2 (moderate), and 3 (severe), each represent a specific hypoxemia severity score. They are based on the NEWS2+ scoring system in respect to the Spo2, age, and type of disease (see feature engineering, Section 2.3.1).

- **Correlation Analysis:** We generated correlation matrices to assess relationships between physiological variables (Figure 5).

### 2.5. Principal Component Analysis (PCA)

**Figure 6:**
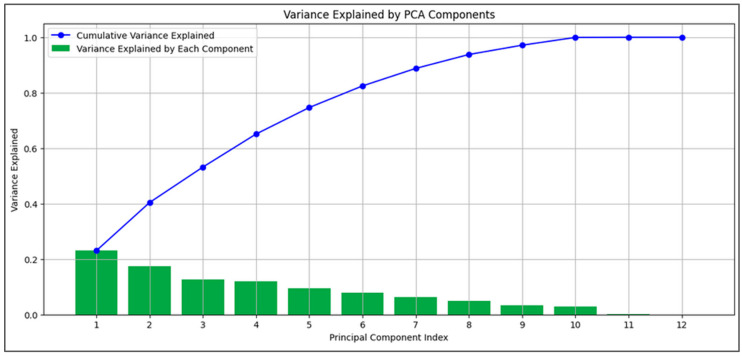
**PCA variance explained and feature contributions.** In green, the percentage of variance accounted for by each principal component is illustrated, and, in blue, the cumulative variance of each PC added to those before.

PCA was performed after imputation and interpolation to assess the variance explained by each principal component and feature contributions. Due to PCA’s sensitivity to data scaling, we applied standardization beforehand. The cumulative variance from the first few components indicated that a reduced feature set could capture most data variability (Figure 6). We focused on physiological variables and easily obtainable features (e.g., gender, height, weight) relevant to CBRNE disaster scenarios, excluding other features for this analysis. While PCA is a linear technique and medical data can show non-linear properties, the results provide useful insights into our physiological features, though they should be interpreted cautiously.

### 2.6. Model Development

#### 2.6.1. Model Selection and Rationale

We selected six models for comparison: Gradient Boosting Models (XGBoost [34], LightGBM [32], CatBoost [33]), Random Forest (RF) [52], and sequential models (LSTM [37] and GRU [36]). The choice was motivated by recent studies demonstrating the effectiveness of both GBMs and sequential models in handling medical time series data [53,54,55,56,57]. RF was chosen as a good baseline among the ensemble models.

Gradient Boosting Models, like XGBoost, excel in medical diagnostics, achieving high accuracy (e.g., 83% in heart failure survival predictions [58,59]), while providing feature importance insights, such as identifying “follow-up time period” is key [59]. They handle mixed data types [60], are robust to overfitting [58], and were included to leverage their strengths and compare with other strong models for potential differences. Random Forests handle high-dimensional datasets, support feature selection to identify key variables, and are robust to outliers, making them suitable for diverse medical data [58,59]. Voting classifiers, which combine multiple models, were included to further enhance robustness and generalization. By reducing bias through the combination of algorithms, they can improve generalization to unseen data—crucial in medical diagnostics with high patient variability—and can be less affected by the weaknesses of individual models, leading to more reliable predictions [59].

Deep learning models, particularly LSTMs, excel in sequential medical data analysis, achieving good results in blood oxygen saturation prediction [61]. The Dipole model, using bidirectional RNNs, effectively predicted future health from Electronic Health Records (EHRs) data by capturing temporality and long-term dependencies [62]. RNN variants further demonstrate promise in medical applications. A hybrid LSTM-GRU model detected Parkinson’s disease from voice recordings [63], while an attention-based RNN framework improved multi-diagnosis predictions by modeling EHRs data temporality [64].

Gradient Boosting Models (GBMs), specifically Gradient Boosting Decision Trees (GBDT), and Random Forests (RF) are highly effective for tabular data, as they can model complex, non-linear relationships. These ensemble methods, based on decision trees, operate differently: GBMs use boosting to sequentially correct errors in predictions, while RF employs bagging, training multiple models independently on bootstrap samples (random subsets with replacement). RF aggregates predictions through voting (for classification) or averaging (for regression), effectively reducing variance and mitigating overfitting by leveraging diverse data subsets. Notably, GBMs and RF do not require standardization or normalization of input features, as their decision-making relies on threshold values learned during training. This characteristic enhances their practicality for medical applications, where data often exhibit wide-ranging scales and distributions.

The Sequential Models are designed to capture temporal dependencies in sequential data. Using medical time series data, we applied these models to investigate and compare the performance of deep learning approaches (sequential models) with “simpler” machine learning methods, specifically the four tree-based models mentioned above.

#### 2.6.2. Gradient Boosting Models (GBMs) and Random Forest (RF)

We implemented Random Forest, XGBoost, LightGBM, and CatBoost classifiers. Their characteristics are presented in Table 7 below. They offer advantages such as handling missing values and providing feature importance see Section 3.4.1. Note that the shift lag process for the labels was applied for RF, GBMs, and sequential models (see experimental setup at Section 2.6.4 for the shift lag part and further explanations).

Each GBM offers unique features designed to address specific modeling challenges. For instance, XGBoost applies early stopping and class weights to address class imbalance, CatBoost handles categorical variables effectively and reduces prediction shifts, and LightGBM is optimized for speed with Gradient-based One-Side Sampling.

#### 2.6.3. Sequential Models

After the preprocessing steps described in Section 2.3, the data retained its original tabular format. It was then processed differently for sequential models via their respective Dataloader, while remaining unchanged for the GBMs and RF.

We implemented masked LSTM and Masked GRU models to process sequential data, addressing variable sequence lengths through padding and the masking process.

More specifically, among the 2169 unique admissions in the three datasets, sequence lengths—representing the duration of each admission—ranged from 952 to 254,643 rows, with a median of 5941 rows and an average of 10,417 rows (each row corresponding to one minute per admission). Thus, a significant difference in terms of the length.

To ensure computational efficiency of the sequential models, we standardized the sequence length to 1024 rows—about 18 h per admission. Longer sequences were split into 1024-row segments, while shorter ones were padded with a constant value of 1000, which did not overlap with any actual feature values. This preprocessing generated 23,099 admissions suitable for sequential models.

The masked LSTM and GRU models were designed to ignore padded values (set to 1000) using an internal mask within the Dataloader. This mask excludes padded values in sequential input data, ensuring they do not influence model training. Notably, this type of “masking” differs from the masks used to identify imputed or interpolated (synthetic) data (see Section 2.3.2).

Variable-length sequences are effectively managed through masking and sequence packing. Sequence lengths are calculated from the mask, and sequences are sorted by length. Using PyTorch’s pack_padded_sequence, sequences are packed so that LSTM/GRU layers only process valid time steps, omitting padding from computations. After processing, sequences are unpacked and returned to their original order, enabling efficient batch processing of variable-length sequences and ensuring that padding does not interfere with the model’s learning.

**Figure 7:**
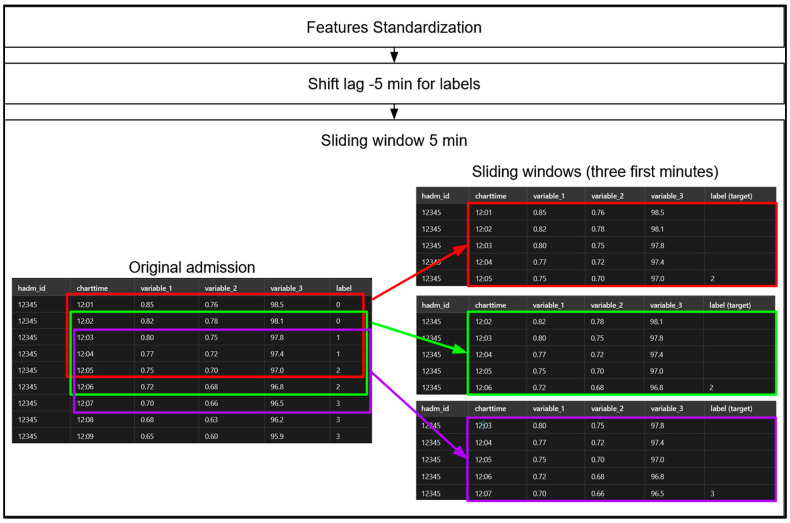
**Overview of main preprocessing steps for sequential models with a 5-min sliding window illustration for the initial minutes of admission. Step 1**: involves standardizing the data using Scikit-Learn’s StandardScaler, but only for our sequential models. Tree-based models, such as gradient-boosted machines (GBMs) and Random Forest (RF), operate based on threshold values; therefore, standardization is unnecessary for them, and likewise for the voting classifier ensemble. **Step 2**: we apply a −5-min shift to the label values, pairing each row with the label occurring 5 min later. This adjustment allows each row, representing a set of physiological measurements at a specific minute of a patient’s admission, to serve as the input for predicting the hypoxemia severity score 5 min in advance. Notably, this label-shifting process is performed for the sequential models, RF and GBMs. **Step 3**: we create 5-min sliding windows for our sequential models only, as GBMs and RF do not leverage temporal sequences. Each sequential model, therefore, considers a 5-min sequence of admission data at a time to predict the hypoxemia severity score label 5 min ahead. We then “jump” forward by one row (or one minute), creating a 4-min overlap between consecutive windows, and repeat this process until we reach the end of the data for each patient admission. This iterative process, carried out in the dataloader of our sequential models, is illustrated here using colors for the first minutes. The red section represents the first 5 min of an admission (from 12:01 to 12:05), which is taken as input for the model to predict the output label “2”. Afterward, a row (or one minute) is skipped to select the next 5-min segment, shown in green (from 12:02 to 12:06), to predict the label of the associated 5th minute of this segment and again produces the label “2”. Subsequently, another row is skipped to obtain the next 5-min segment, displayed in purple (from 12:03 to 12:07), to predict the score of the associated 5th minute of this segment, which is “3”.

See Figure 7 for an overview of the preprocessing steps for the sequential models.

Table 8 below offers a clear overview at a glance by summarizing all the features in a concise format. It includes the features, their descriptions, their types (explained in the legend), and their respective measurement units.

#### 2.6.4. Experiments’ Setup

To prevent data leakage, we ensured that each patient’s data appeared in only one of the training, validation, or testing sets. Admissions were split accordingly.

**Figure 8:**
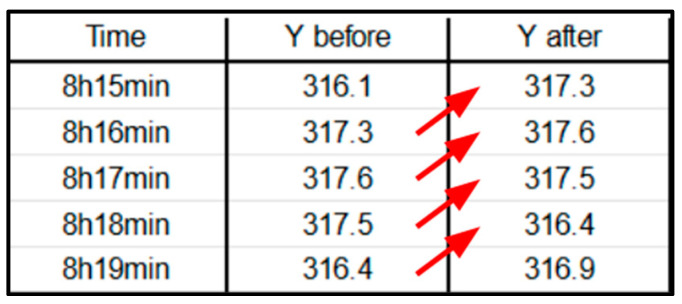
**Shift-lag illustration for a shift of −1 (for illustrative purposes).** Observe the red arrows to see how each y label is moved one position backward (denoted as −1). The result: the label of each given row is the future severity in x-minutes (where label “Y” represents, in our case, the hypoxemia severity score).

- **Shift-Lag Method:** Applied to predict hypoxemia severity scores 5 min in advance, providing a practical window for medical intervention (Figure 8). Indeed, in order to predict hypoxemia severity scores in advance, we apply a −5-min shift to the label values, aligning each row with the label observed 5 min later. This adjustment allows each row, containing physiological measurements recorded at a specific minute of a patient’s admission, to be used as input for predicting the hypoxemia severity score 5 min ahead. Importantly, this label-shifting technique is consistently implemented for both sequential models, Random Forest (RF) and gradient-boosting machines (GBMs).
- **Class Imbalance Handling:** Computed class weights inversely proportional to class frequencies.
- **Hyperparameter Tuning of the GBMs and Random Forest (RF):** We used the HyperOpt library, a popular tool for hyperparameter optimization, with two objective functions—AUC-based and log loss-based. HyperOpt employs the Tree-structured Parzen Estimator (TPE), a Bayesian optimization technique that iteratively updates a probabilistic model based on prior evaluations and performance metrics. Unlike traditional methods that treat these evaluations as independent, TPE refines its model progressively to capture the relationship between hyperparameters and performance, enhancing optimization efficiency.
  ○ **AUC-Based**: This function focuses on maximizing the AUC score by minimizing 1 − AUC, which is especially beneficial when dealing with class imbalance.
  ○ **Log Loss**: This function minimizes log loss (cross-entropy loss), assessing how closely the predicted probabilities match the actual labels, and penalizing incorrect or overly confident predictions.

## 3. Results

### 3.1. Dataset Characteristics

After preprocessing, the dataset consisted of 22,595,035 rows and over 40 feature columns, encompassing both original and engineered features. These features included physiological variables, NEWS+ derived scores (TAGs), demographic data, and mask features (the list of features is available in Table 8).

Each admission was interpolated to minute-level intervals, resulting in a substantial increase in data volume. The length of admissions varied, ranging from approximately 16 h to 177 days for the GBMs and RF. However, for the sequential models, all admission durations were standardized to approximately 16 h by padding shorter admissions and truncating longer ones.

### 3.2. Imputation and Interpolation Outcomes

Different imputation strategies were compared. The histogram-based gradient boosting imputation yielded satisfactory results and was selected for further analysis. Imputations reduced the percentage of missing values to zero, enabling subsequent interpolation at the minute level.

Linear interpolation was chosen over polynomial and cubic spline interpolations due to its ability to produce plausible physiological values (Table 3). Polynomial and cubic spline interpolations resulted in implausible negative values and incorrect magnitudes (see Section 2.3.2).

### 3.3. Exploratory Data Analysis Findings

The dataset exhibited an inherent imbalance, with the distribution of severity scores remaining consistent both before and after interpolation. More severe scores were less frequent, reflecting this imbalance. In terms of label durations, severity score 2 had the shortest median duration of 29 min, while severity score 0 had the longest median duration, lasting 178 min.

### 3.4. Model Performance

#### 3.4.1. Tree-Based Models’ Results and Discussion

The GBMs and RF demonstrated good performance in predicting hypoxemia severity. Hyperparameter tuning yielded only slight improvements over baseline models.

**Training and Convergence:**

**Figure A1:**
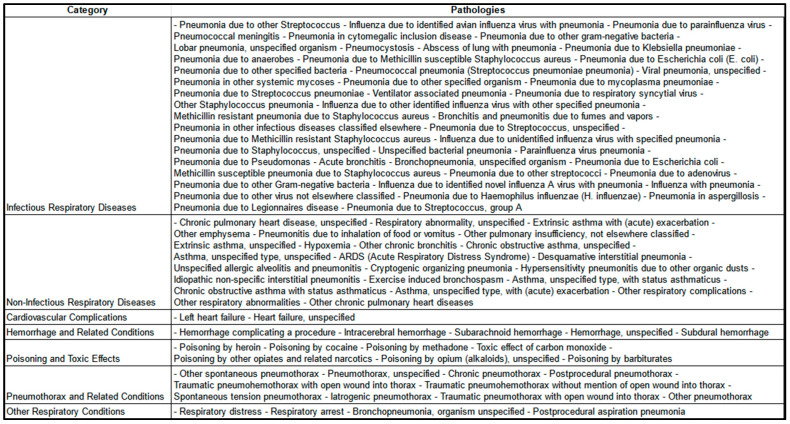
**Selected pathologies in the admissions**: the pathologies were associated with low blood oxygenation levels, chosen to reflect the desired patient-type distribution. These remaining pathologies among admissions, manually selected by medical doctors, are represented here in an arbitrary manner for quick reading overview.

All the tree-based models converged rapidly, with XGBoost models converging in fewer than 65 iterations. CatBoost and LightGBM showed good generalization, sometimes performing better on the validation set. Random Forests (RFs) required slightly more training time than Gradient Boosting Machines (GBMs), as RF involves training 100 estimators for bagging (see Section 3.4.3: comparison between GBMs and sequential models). The performance of RF was comparable to that of GBMs and, in some cases, even outperformed GBMs on certain metrics (see Appendix A Figure A1).

**Best Hyperparameters:**

We used the HyperOpt library for hyperparameter optimization with two objective functions: AUC-based and log loss-based. HyperOpt utilizes the Tree-structured Parzen Estimator (TPE), a Bayesian optimization method. Details of the experimental setup are in Section 2.6.4. Table 9 summarizes the best objective functions, and corresponding hyperparameter values.

**Features Ablation Study:**

**Figure 9:**
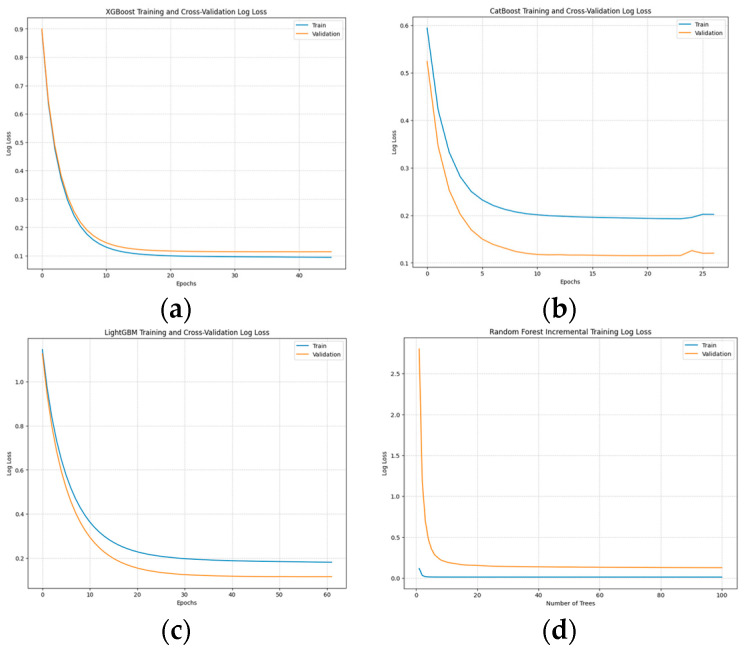
**Training curves of baseline models showing loss over iterations for the 5 min in advance predictions.** XGBoost (**a**), CatBoost (**b**), LightGBM (**c**), and RF (**d**). XGBoost showed slight overfitting, while CatBoost and LightGBM generally demonstrated better generalization to the validation set com-pared to the training set. RF displays some overfitting. GBMs are trained iteratively over successive epochs, with each new tree improving the performance of the previous ensemble. In contrast, RF trains multiple independent trees using bagging, which explains the difference observed along the *x*-axis. Given the overall good performance of all models, we concluded that they all learned ef-fectively during training. Notice that to plot the Random Forest training curves, we trained the RF incrementally for illustration purposes, which, as verified, does not affect its final performance.

To analyze feature importance, we conducted an ablation study on the baseline XGBoost models by removing the top two most important features: SpO_2_ and heart rate. This experiment revealed significant changes in model performance and learning dynamics. Specifically, the training curves exhibited both underfitting and overfitting tendencies, with increased train and validation loss values. The validation loss plateaued at 0.32, while the training loss reached 0.25, both higher than the desired convergence around 0.1 (refer to Figure 9).

Additionally, removing these features altered the feature importance rankings, as evidenced by changes in the relative ranks of the remaining features. This shift highlights a disruption in the overall learning process.

Performance metrics were also adversely affected. Sensitivity (recall) dropped to 0.45, 0.44, and 0.36 for classes 1, 2, and 3, respectively. Specificity experienced a less pronounced decline. The Matthews Correlation Coefficient (MCC) fell sharply from 0.89 to 0.65 (refer to Section 3.4.1 in performance metrics part). Both the ROC and PR curves showed degradation, with the PR curves being particularly impacted: values for classes 1, 2, and 3 dropped to 0.66, 0.24, and 0.42, respectively (likewise, see Section 3.4.1 and Appendix A Figure A1 for details without ablation).

These results highlight the critical importance of SpO_2_ and heart rate in the XGBoost model’s training and prediction processes, illustrating how dependence on specific features impacts model performance and stability. This finding reinforces our assumption that meaningful patterns exist within the data and underscores the essential role of diverse features in characterizing it.

**Confusion Matrices:**

The observations described in this section apply to both TBM and sequential models. For detailed confusion matrices, with hypoxemia severity scores as labels, refer to Appendix A Figure A6 and Section 3.4.2. Labels 2 and 3 were frequently misclassified, indicating challenges in predicting transitions between severity levels. Label 0 was most accurately predicted, followed by labels 3, 1, and 2 (see label distribution, Section 2.4 and Table 5). This aligns with the inherent challenge of predicting events that are both sudden and rare, such as label “2”. Importantly, this indicates that we can predict our primary complication, label 3, which corresponds to the most severe hypoxemic condition, with a reasonable degree of accuracy, supporting our proof of concept (POC).

**Feature Importance and Model Interpretability:**

**Figure 10:**
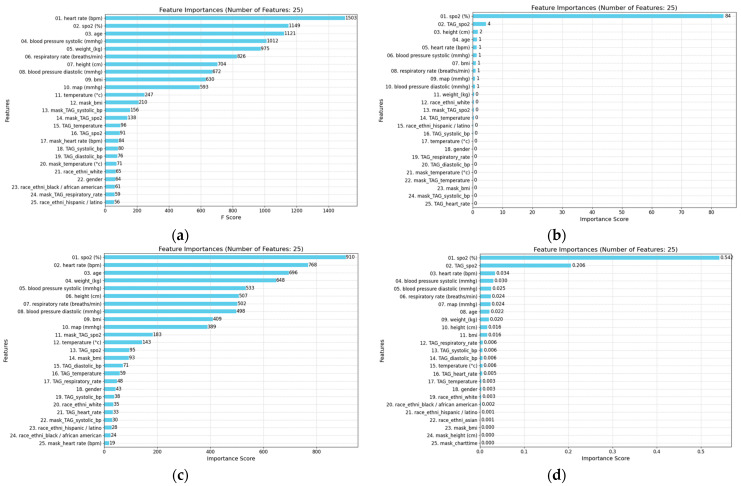
**Top 25 feature importance derived from the baseline GBMs and RF for predicting hypoxemia score 5 min in advance:** XGBoost (**a**), CatBoost (**b**), LightGBM (**c**), and RF (**d**). The models do not utilize the features in the same way when constructing their successive prediction trees. This dis-crepancy highlights the differences in how each model prioritizes and processes the various features to make predictions, as well as the fundamental differences introduced by the learning techniques of boosting and bagging.

The tree-based models offer advantages such as providing feature importance (see Figure 10). Our feature importance analysis sheds light on the key determinants of model performance, emphasizing the significance of physiological variables and TAG features while also revealing the varying relevance of mask features across different algorithms. These findings enhance the interpretability of machine learning predictions in clinical contexts and open pathways for addressing potential biases linked to demographic factors.

We conducted a thorough examination of feature importance across multiple machine learning models, including Gradient Boosting Machines (XGBoost, LightGBM, CatBoost) and Random Forests (RFs). This analysis focused on the contributions of physiological variables, TAG features from the NEWS2+ system, and mask features indicating synthetic data presence. Notably, mask features were excluded from CatBoost models.

The results highlighted that for XGBoost and LightGBM models, physiological variables like heart rate, systolic blood pressure, age, weight, and respiratory rate were the most impactful (see Figure 10). For CatBoost, TAG features such as SpO_2_, TAG SpO_2_, height, age, and heart rate took precedence, with mask features playing a negligible role (see Figure 10). Across all models, SpO_2_ levels, heart rate, age, and respiratory rate consistently emerged as top predictors, aligning with clinical expectations and demonstrating the models’ ability to leverage inherent patterns in physiological data effectively.

Mask features were highly valued by XGBoost and LightGBM, reflecting these models’ ability to distinguish real data from synthetic data generated through imputations and interpolations, corroborating prior research [42,43,44,45]. Conversely, CatBoost and RF models disregarded mask features, demonstrating variability in feature utilization across algorithms.

TAG features proved to be consistently important, reaffirming the predictive strength of the NEWS2+ system’s scoring methodology. While demographic factors such as race and gender were less influential overall, their appearance among the top 25 features in some GBM and RF models highlights the need for ongoing efforts to detect and mitigate potential biases in model predictions.

**Performance Comparison of Ensemble Voting Classifiers with Tree-Based Models:**

The primary goal of this comparison was to evaluate the potential benefits and trade-offs of ensemble methods and provide insights to guide future research, particularly in improving temporal modeling and generalizability.

We employed Voting Classifier models from the Scikit-learn library to construct ensemble models comprising multiple tree-based methods (ensembles of ensembles). These Voting Classifier ensembles consisted of either three GBMs (Gradient Boosting Machines: XGBoost, CatBoost, and LightGBM) or four models, which included the same GBMs with the addition of a Random Forest (RF). All tree-based models in the ensembles were the baseline models used previously, with hyperparameters detailed in Table 6 (Section 2.6.2). Soft voting, which averages predicted probabilities to select the class with the highest average, and hard voting, which applies a majority rule to predicted class labels, were implemented for each ensemble. Training times ranged from 11 min for GBMs-only ensembles to 20 min for those including RF.

From a coding perspective, implementing the VotingClassifier required an alignment of functionalities and minor adjustments. For example, XGBoost’s *objective* parameter needed to match the voting method (*multi:softprob* for soft voting and *multi:softmax* for hard voting). Soft voting relied on *predict_proba* for class probabilities, while hard voting was used to *predict* class labels. Output consistency in shape and format across all models was crucial, requiring adjustments for CatBoost. Fitted estimators were accessed using *voting_clf.named_estimators_* (see Appendix A).

**Figure A3:**
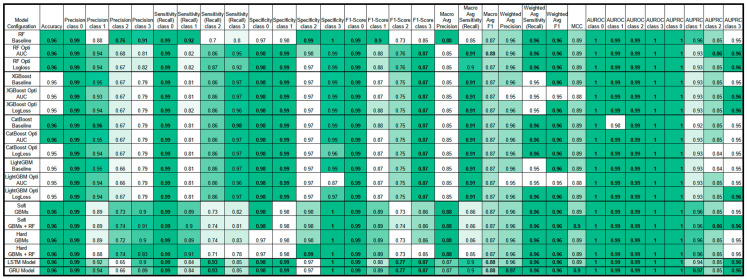
**Detailed comparison table of the performance metrics for Gradient Boosting Models (XGBoost, CatBoost, LightGBM), RF, Sequential Models (LSTM, GRU), and Voting Classifier ensembles under various configurations.** Both sequential models display competitive performance, high-lighting their suitability for time-series classification tasks alongside GBMs and RF, though they require significantly longer training times. Optimizing GBMs and RF with HyperOpt via the ob-jective functions (AUC and LogLoss) provides slight improvements, underscoring the impact of hyperparameter tuning on the performance. The Voting Classifier ensembles show slight im-provements in some metrics but exhibit a precision-sensitivity tradeoff, with a slightly lower F1-score for severity levels 2 and 3. Note that the metrics are reported with varying decimal preci-sion; however, this does not indicate any advantage for models with more precise values. The goal is simply to provide a quick overview of performance results for comparison and exploration in this proof of concept. Despite these variations, the table offers a comprehensive overview to support analysis and interpretation. For better visualization, a darker green shade in each metric column corresponds to a higher number.

Overall, soft voting consistently delivered slightly better performance than hard voting, and ensembles combining GBMs and RF outperformed those consisting solely of GBMs, especially with soft voting (see Appendix A Figure A3 and performance metrics part below). Precision was higher for classes 2 and 3 with ensembles, while precision and sensitivity for class 0 remained consistent across all machine and deep learning models. However, precision for class 1 was generally lower for ensembles, though sensitivity was higher for this class. Sensitivity for classes 2 and 3 saw slight reductions. F1-score differences were minor, with slight improvements for classes 0 and 1 and marginal decreases for classes 2 and 3. These decreases are less favorable, as classes 2 and 3—particularly class 3—are critical in this use case.

The AUROC values for all classes remained consistent at 1, 0.99, 0.99, and 1 for classes 0, 1, 2, and 3, respectively. The AUPRC scores for class 0 were consistently 1 across all models. For other classes, the AUPRC scores were slightly higher for the ensembles, particularly for classes 1 and 2. The AUPRC for class 1 exceeded those of individual tree-based models, except for the RF baseline model, which achieved the same value (see Appendix A Figure A3 and performance metrics part below).

The highest MCC score of 0.9 was achieved by the GBMs + RF ensemble with soft voting, equaling the performance of the GRU model. Specificity for classes 2 and 3 was generally higher with ensembles, with class 3—representing urgent cases—achieving a perfect specificity of 1, comparable to sequential models. However, specificity for classes 0 and 1 was marginally lower, which is less critical in practical applications due to the lower severity associated with these classes.

**Figure A4:**
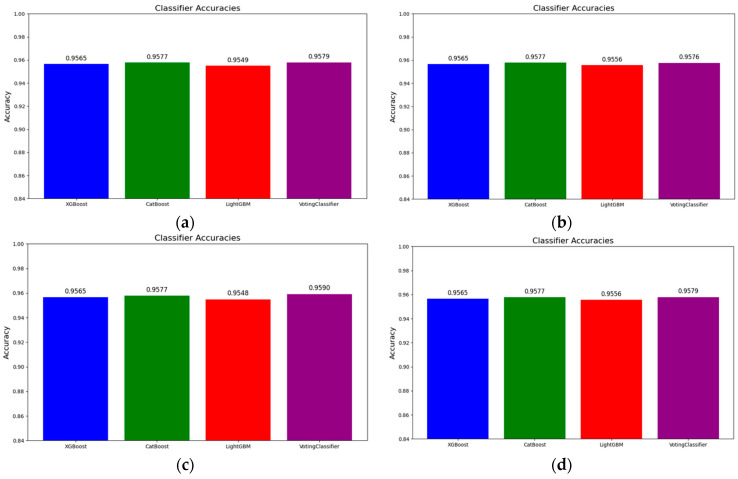
**Comparison of accuracy scores for XGBoost, CatBoost, LightGBM, and ensemble Voting Classifiers.** We visualize accuracies to assess how ensembles improve over individual models, identify the best-performing classifier, and compare ensemble performance against individual models. The Voting Classifiers combine either the three GBMs (XGBoost, CatBoost, LightGBM) or the three GBMs + RF, using soft or hard voting methods. Accuracy values are evaluated on a test dataset and displayed within the 85–100% range for clarity. Bars are annotated with accuracy scores to enhance interpretability. (**b**)—soft voting (3 GBMs), (**a**)—hard voting (3 GBMs), (**c**)—soft voting (3 GBMs + RF), (**d**)—hard voting (3 GBMs + RF). The voting classifier ensemble shows only slight accuracy improvements compared to the individual baseline models.

**Figure A5:**
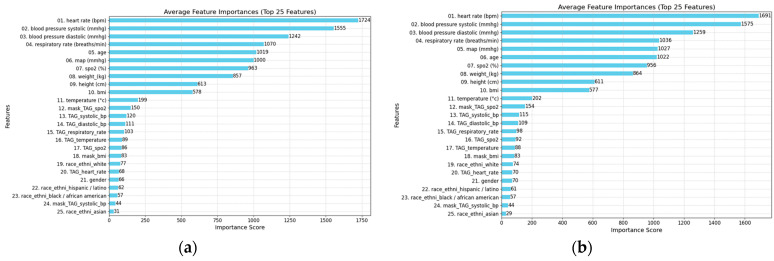

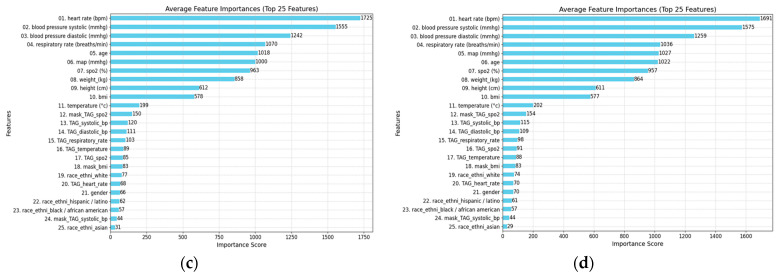
**Top 25 Feature Importance for ensemble Voting Classifiers.** (**b**)—soft voting (3 GBMs), (**a**)—hard voting (3 GBMs), (**c**)—soft voting (3 GBMs + RF), (**d**)—hard voting (3 GBMs + RF).

**Figure A6:**
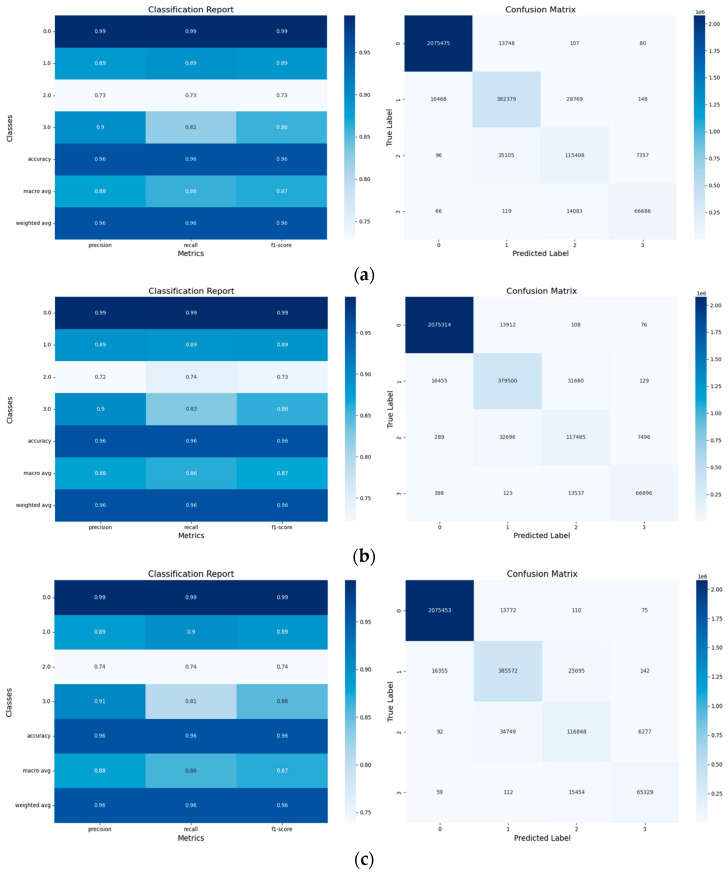

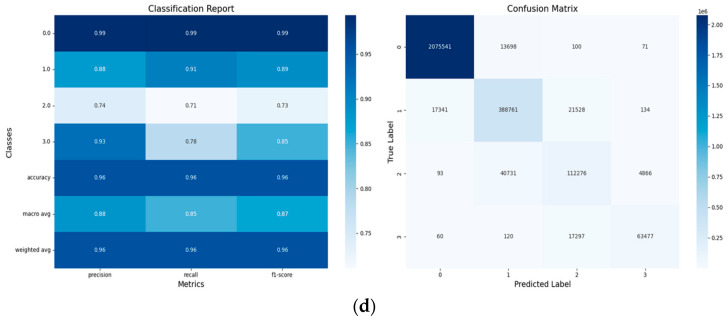
Confusion matrices and classification reports for ensemble Voting Classifiers. (**a**)—soft voting (3 GBMs), (**b**)—hard voting (3 GBMs), (**c**)—soft voting (3 GBMs + RF), (**d**)—hard voting (3 GBMs + RF).

**Figure A7:**
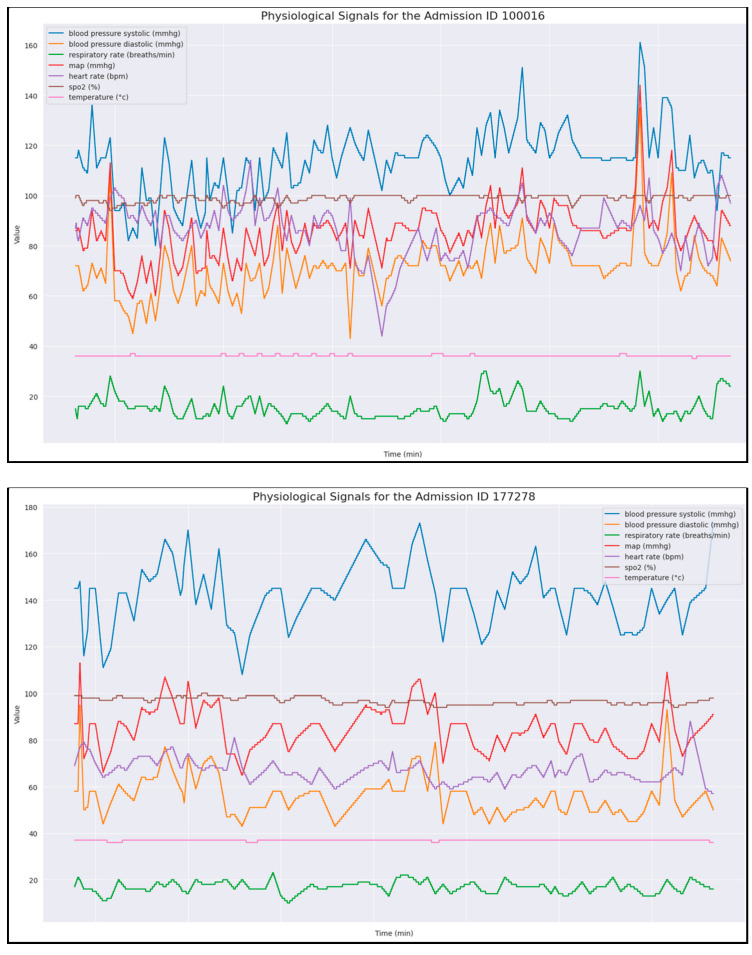
Physiological measurements during admissions: Variation in seven physiological variables is illustrated after minute-by-minute interpolation, with prior imputation using Histogram-based Gradient Boosting.

In conclusion, Voting Classifiers composed of tree-based models with default parameters did not yield significant performance improvements over individually optimized models (see also Appendix A Figure A4, Figure A5 and Figure A6 for more detailed analysis). Nonetheless, slight enhancements were observed for certain metrics. The choice of method depends on the specific use case, whether prioritizing precision or sensitivity, alongside F1-scores for key classes, specificity, and other metrics. Finally, it is important to note that metric differences were often minor and limited to rounding effects, as this analysis was intended as a proof of concept rather than precise optimization. Therefore, results should be interpreted cautiously, although observed trends highlight promising directions for future research. The Voting Classifier ensemble could serve as a viable compromise to sequential models, which require significantly longer training times, in future research.

**Performance Metrics:**

Table 10 below provides a performance benchmark for the best-fine-tuned models and the Voting Classifier ensembles. Notably, for all three GBMs, performance differences between each fine-tuned model and its respective baseline (model with default hyperparameters) were minimal, with metrics remaining closely aligned. Voting Classifier ensembles showed only slight enhancements.

#### 3.4.2. Sequential Models’ Results and Discussion

The LSTM and GRU models were trained using a 5-min sliding window, with padding and masking applied to handle variable sequence lengths (see Section 2.6.3). By saving the models’ parameters and hyperparameters at each epoch, we were able to reload the model from the optimal training epoch, ensuring that the best performance was retained post-training.

**Performance Metrics:**

We compared the performance for both sequential models (Table 11).

**Training Time:**

**Figure 11:**
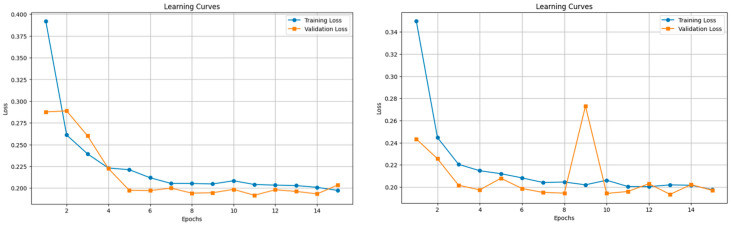
Training curves over epochs for LSTM (**left**) and GRU (**right**) models.

Training time was significantly longer than GBMs, RF, or Voting Classifier ensembles. Each training epoch took approximately 2.5 h. Total training time was around 37.5 h for each sequential model in the Figure 11.

**Figure 12:**
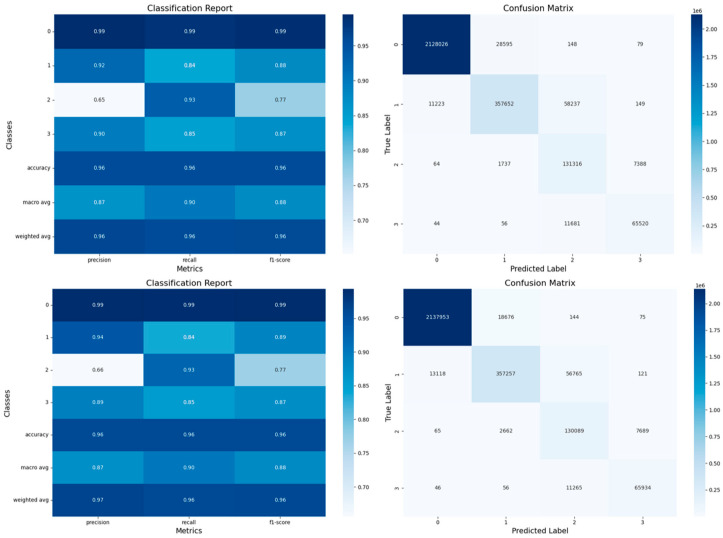
**Confusion matrices and classification reports for LSTM and GRU models for the test set.** The LSTM results are displayed at the **top**, and the GRU results at the **bottom**.

Observations derived from Figure 12 apply to both TBM and sequential models, with label 2 frequently misclassified due to rarity, while label 3 (severe hypoxemia) is predicted accurately, supporting the POC.

#### 3.4.3. Comparison Between GBMs, RF, and Sequential Models

While sequential models showed marginal improvements in some metrics, the gains did not justify the significantly longer training times and increased computational resources.

Concerning interpretability, GBMs and RF offer greater transparency through feature importance metrics. Regarding training efficiency, GBMs trained much faster than sequential models (less than 3 min vs. 37.5 h, per model). Random Forests (RFs) required more training time (around 14 min) than GBMs due to the need to train a certain number of estimators for bagging. Therefore, we would advocate further for the use of GBMs in model training.

Finally, for the performance trade-offs, sequential models performed generally slightly better but are less practical for rapid deployment.

**Performance Metrics:**

Table 12 provides a performance comparison between GBMs and sequential models. For a fairer assessment, we include the best fine-tuned GBM models optimized via HyperOpt TPE, alongside the performance results of the sequential models.

## 4. Discussion

Our study demonstrates that tree-based machine learning models, particularly gradient boosting machines (GBMs) and Random Forest (RF), effectively predict hypoxemia severity scores using physiological parameters, among other factors. The strong performance of GBMs and RF suggests that temporal data may not be essential for this prediction task. This aligns with findings from other studies indicating that deep learning models may not be ideal for tabular data, which, in our case, specifically takes the form of a time series [65]. It is likely that boosting, or, alternatively, bootstrapping combined with bagging, constitutes sufficient learning techniques.

**Key Findings:**

In comparing tree-based and sequential models, GBMs and RF offer a practical balance of performance, interpretability, and training efficiency, while sequential models yield only slight performance gains at a much higher computational cost, especially when compared to the optimized RF model. Both types show strong, competitive performance in multi-class classification, though with different strengths and limitations. Tree-based models, while not designed to capture temporal dependencies, provide competitive results and train quickly, making them highly efficient (in particular GBMs). In contrast, sequential models, which consider temporal information, take significantly longer to train. For the purposes of this study, they were not fine-tuned due to time constraints, though further tuning might improve their performance.

In terms of feature importance, the inclusion of mask and TAG features notably improved model performance, emphasizing the value of transparent data preprocessing. Additionally, the TAG features derived from the NEWS2+ system demonstrated innovation, proving highly effective in enhancing model accuracy. These observations align with the findings of the feature ablation study (see Section 3.4.1), where the removal of highly ranked features, in the feature importance hierarchy, resulted in a negative impact on model performance. This reaffirms that each of these ranked features is essential to the model’s predictive capability.

Regarding class imbalance, the class-weighted strategy allowed the models to handle the unbalanced dataset effectively, particularly in predicting the most severe hypoxemia cases (label 3). Even though label 1 was more prevalent, the models predicted label 3 accurately, showcasing the success of the class-weighted approach.

The balance between sensitivity and specificity in model performance is challenging [28], especially for medical event predictions. High specificity with low sensitivity may reduce unnecessary interventions and related costs, as seen with D-dimer testing for venous thromboembolism [66], but could miss critical cases due to missing relevant variables or a limited number of outcome events, making such models more suited as a decision support tool rather than a stand-alone diagnostic tool. Conversely, high sensitivity with low specificity might capture non-specific patterns—such as variables associated with hypoxia but not exclusive to it—or detect subtle changes in nonhypoxic cases, leading to excessive false alerts and limiting clinical usability.

**Limitations and Perspectives:**

The data for this study were sourced from the MIMIC-III and IV databases, which may limit the generalizability of the findings. Our preprocessing involved minute-by-minute interpolation; however, it is important to note that the data distribution from MIMIC-III and IV reflects a single hospital with no pediatric data (the minimum age post-preprocessing is 12–17 years). We also stratified patient ages into categories (see Table 4), but due to the dataset’s lack of pediatric data and its imbalance across race and ethnicity categories, the model’s real deployment potential is limited with respect to these critical demographic factors.

Additionally, while the SpO_2_/FiO_2_ ratio (FiO_2_ represents the Fraction of Inspired Oxygen in the inhaled gas) is a common indicator of respiratory distress in both clinical and pre-hospital settings (e.g., medical evacuation) [67,68], our study takes a different approach due to limitations in the available data. The MIMIC databases lack sufficient FiO_2_ measurements, with a high proportion of values missing. Relying on the SpO_2_/FiO_2_ ratio would have introduced excessive uncertainty into the classification; instead, we prioritize SpO_2_ measurements, which provide a more reliable basis for categorization. Imputing FiO_2_ data to generate an SpO_2_/FiO_2_ ratio would have likely introduced further biases, especially in instances where both SpO_2_ and FiO_2_ values required imputation.

In terms of temporal information, sequential models (e.g., LSTM, GRU) did not demonstrate a significant performance advantage over GBMs and RF, indicating that temporal dependencies may not be essential for predicting hypoxemia severity in this context. Nonetheless, to further validate our study, we should consider using a dataset with naturally regular intervals and fewer missing values, such as HiRID [69], which provides continuous physiological values recorded every two minutes, reducing the need for interpolation and imputation.

The Voting Classifier ensembles showed only small performance gains over individually optimized models, with trade-offs in precision and sensitivity, especially for critical classes. Improvements in one metric sometimes came at the expense of another, underscoring the need for balance. Small metric differences, possibly due to rounding, limit generalizability but offer valuable insights for future applications. These ensembles balance computational efficiency and performance, being faster than sequential models while offering small improvements over individual GBMs or RFs in some cases, albeit with slightly longer training times. Future research should focus on hyperparameter optimization and feature engineering to maximize their potential. With refinement, Voting Classifier ensembles could become reliable and robust solutions for various machine learning tasks, balancing efficiency and performance effectively.

**Implications for Practice:**

GBMs can be integrated into systems like the VIMY Multi-System for real-time hypoxemia severity prediction, enabling rapid deployment in (pre)-clinical settings. Similarly, RF models can be considered for integration, provided they execute quickly enough. Understanding the correlation between features and their importance is crucial for both model performance and interpretability. The feature importance metrics provide valuable insights for clinicians, aiding in the understanding of model decisions and improving trust and adoption, which ultimately enhances the interpretability of the system.

Additionally, we have a highly heterogeneous population, which is advantageous as our goal is to predict hypoxia in CBRNE situations in a broadly applicable, non-specific manner. A diverse population supports this aim, increasing the likelihood of developing a model with wide-ranging applicability.

**Future Work:**

Several key areas will be explored to enhance the performance and applicability of the models. First, data integration will focus on incorporating data from multiple hospital databases to improve the model’s generalizability across diverse populations. The algorithms will then be tested prospectively on critically ill patients before being deployed in pre-clinical settings. Bias mitigation will ensure that the models remain free from biases related to race and gender, promoting fairness in outcomes. Lastly, real-time implementation will involve developing efficient algorithms suitable for deployment in resource-constrained environments, enabling timely and effective predictions in practical settings.

Fine-tuning the sequential models, despite the training time constraints, could likely bring even minor improvements, which in medical settings can be impactful. We also plan to test the recent xLSTM model, known for its efficiency and lighter architecture compared to other attention-based models, to assess its suitability in this context.

## 5. Conclusions

This study highlights the potential of machine learning models, particularly tree-based approaches, in predicting hypoxemia severity five minutes in advance for disaster triage scenarios (see Figure 1 and Figure 2). Among the models evaluated, gradient boosting machines (GBMs) demonstrated robust performance and practicality for integration into systems such as the VIMY Multi-System. Their efficiency, marked by fast training times, makes them ideal for real-world applications and validates the effectiveness of boosting strategies. Meanwhile, Random Forests (RFs) achieved the best overall performance among tree-based models, despite requiring longer training times. This suggests that bootstrapping, especially when combined with bagging (as in RF), is a highly effective strategy for this task, even without the use of boosting.

The Voting Classifier ensembles, composed of tree-based models with baseline parameters, showed slight improvements in some metrics but faced a precision-sensitivity tradeoff, with a slightly lower F1-score for severity levels 2 and 3 (see Table 10 and Section 3.4.1). The best ensemble combines XGBoost, CatBoost, LightGBM, and RF using a soft voting method during fitting (see Appendix A Figure A3 and Figure A4 and Table 12).

This study addressed challenges related to noisy or incomplete data by applying masking techniques, imputation, and interpolation methods to establish regular time intervals without missing data, ensuring readiness for model training. Importantly, focusing on easily measurable physiological parameters, such as respiratory rate, SpO_2_, and blood pressure, ensures practicality in preclinical CBRNE scenarios. These parameters, critical indicators of respiratory and cardiovascular function, are compatible with wearable devices, enabling seamless data collection and rapid deployment in high-stakes environments.

Notably, temporal dependencies, while theoretically beneficial, were found to be non-critical for predicting hypoxemia severity within the given timeframe. Sequential models, despite their capacity to capture temporal patterns, yielded only marginal improvements that did not justify their higher computational costs. Tree-based models emerged as the optimal choice, balancing computational efficiency, execution speed, and interpretability—essential qualities in resource-constrained scenarios.

A pivotal aspect of this research was the development of the NEWS2+ early warning system, which enabled advanced feature engineering (Table 2). NEWS2+ generated feature scores (TAGs) for six primary physiological parameters and created SpO_2_-based hypoxemia severity scores tailored to patient age and disease type for labeling purposes. These clinically informed labels provided a novel foundation for accurate and relevant severity prediction.

Future efforts will focus on enhancing model generalizability by incorporating broader preclinical datasets to leverage greater demographic and clinical variability. Additionally, plans include integrating these models into real-time monitoring systems to ensure timely interventions and improved patient outcomes in emergency medical scenarios.

This study establishes a foundational proof of concept for advancing predictive modeling in critical scenarios, offering a novel approach to triage that prioritizes clinical relevance, computational efficiency, and practical applicability in preclinical disaster settings.

## Figures and Tables

**Table 1:** **NEWS2+ Scoring Matrices for Hypoxemia Severity by Patient Type.** The NEWS2+ scoring matrices provide a structured approach to determining hypoxemia severity scores across distinct patient groups, specifically tailored for adults without COPD, adults with COPD, and pediatric populations. These scores serve as the labels we aim to predict within our study and are grounded in established research sources [4,8,9,10,11,12,13,17,20,40], with further adaptation by physicians to suit a pre-clinical setting. The matrices provide a patient-type-specific framework for interpreting SpO_2_ thresholds, which are essential for assessing respiratory health. SpO_2_, or oxygen saturation, represents the percentage of oxygenated hemoglobin in the blood, a vital indicator of respiratory function. Each matrix within NEWS2+ is optimized for different oxygenation needs: adults without COPD have specific SpO_2_ targets aligned with general adult respiratory requirements; adults with COPD have adjusted targets reflecting the lower oxygenation needs typical for COPD patients; and pediatric populations have age-specific thresholds to accommodate children’s unique respiratory profiles. Each matrix categorizes SpO_2_ levels into severity classifications to guide clinical assessment and intervention. Severe hypoxemia indicates critically low SpO_2_, requiring immediate medical intervention (class 3). Moderate hypoxemia reflects moderate oxygen depletion, necessitating close monitoring and potential intervention (class 1). Mild hypoxemia signifies slightly low oxygen levels, usually manageable without intensive treatment (class 2). Normal levels denote stable SpO_2_ and healthy respiratory function, not requiring intervention (class 0).

Population	Severe	Moderate	Mild	Normal
Adults without COPD	≤91%	92–93%	94–95%	96–100%
Adults with COPD	≤82%	83–84%	85–87%	88–92%
Pediatric (without COPD)	≤84%	85–88%	89–95%	96–100%

**Table 2:** **NEWS2+ Scoring Matrix: Feature Scores (TAGs) for Physiological Variables in Adults and Pediatric Populations.** The NEWS2+ scoring system is applied to derive feature scores (TAGs) for six primary physiological variables across both adult and pediatric populations. For adults, these feature scores capture key indicators of physiological health, while the pediatric version of NEWS2+ is tailored to accommodate developmental differences across specific age groups. The pediatric matrix is divided into five distinct age groups to ensure age-appropriate scoring: infants under 1 year (0–11 months), toddlers under 2 years (12–23 months), children aged 2 to 4 years, those aged 5 to 11 years, and adolescents aged 12 to 17 years. These age groups are presented sequentially, from top to bottom, in the order listed. This structured approach ensures that the scoring accurately reflects physiological expectations and variations across developmental stages, supporting precise assessment and appropriate clinical responses. In ambient air, for the saturation SpO_2_, the patient is not subject to hyperoxemia so not applicable (N/A).

Age Group	Severe Low (Level 3)	Moderate Low (Level 2)	Mild Low (Level 1)	Normal Beseline (Level 0)	Mild High (Level 1)	Moderate High (Level 2)	Severe High (Level 3)
**Adults**							
Respiratory Rate (BPM)	0–5	6–7	8–9	10–20	21–25	26–29	30–400
Saturation (SpO_2_, %)	0–84	85–88	89–95	96–100	N/A: Ambient Air	N/A: Ambient Air	N/A: Ambient Air
Heart Rate (BPM)	0–40	41–50	51–59	60–90	91–110	111–130	131–400
Systolic BP (mmHg)	0–90	91–100	101–114	115–119	120–129	130–144	145–300
Diastolic BP (mmHg)	0–49	50–59	60–64	65–79	80–85	86–90	91–300
Temperature (°C)	0–35.0	35.1–35.9	36.0–36.6	36.7–37.7	37.8–38.8	38.9–39.8	39.9–60
**0–11 Months**							
Respiratory Rate (BPM)	≤19	20–24	25–29	30–49	50–59	60–69	≥70
Saturation (SpO_2_, %)	≤91	92	93	94–100	N/A: Ambient Air	N/A: Ambient Air	N/A: Ambient Air
Heart Rate (BPM)	≤99	100–104	105–109	110–159	160–164	165–169	≥170
Systolic BP (mmHg)	≤59	60–64	65–69	70–99	100–104	105–109	≥110
Diastolic BP (mmHg)	≤20	19–26	27–37	37–56	57–69	70–89	≥90
Temperature (°C)	≤34.9	35.3–35.4	35.5–35.9	36–37.0	37.1–37.4	37.5–37.9	≥38
**12–23 Months**							
Respiratory Rate (BPM)	≤19	20–22	23–24	25–39	40–49	50–59	≥60
Saturation (SpO_2_, %)	≤91	92	93	94–100	N/A: Ambient Air	N/A: Ambient Air	N/A: Ambient Air
Heart Rate (BPM)	≤79	80–89	90–99	100–149	150–154	155–159	≥160
Systolic BP (mmHg)	≤59	60–64	65–69	70–99	100–104	105–109	≥110
Diastolic BP (mmHg)	≤20	19–35	36–40	41–62	63–70	71–89	≥90
Temperature (°C)	≤34.9	35.3–35.4	35.5–35.9	36–37.0	37.1–37.4	37.5–37.9	≥38
**2–4 Years**							
Respiratory Rate (BPM)	≤14	15–18	17–19	20–34	35–39	40–49	≥50
Saturation (SpO_2_, %)	≤91	92	93	≥94	N/A: Ambient Air	N/A: Ambient Air	N/A: Ambient Air
Heart Rate (BPM)	≤69	70–79	80–89	90–139	140–144	145–149	≥150
Systolic BP (mmHg)	≤69	70–79	N/A	80–99	N/A	100–119	≥120
Diastolic BP (mmHg)	≤18	19–34	35–43	44–67	68–75	76–89	≥90
Temperature (°C)	≤34.9	35.3–35.4	35.5–35.9	36–37.0	37.1–37.4	37.5–37.9	≥38
**5–11 Years**							
Respiratory Rate (BPM)	≤14	15–16	17–19	20–29	30–34	35–39	≥40
Saturation (SpO_2_, %)	≤91	92	93	≥94	N/A: Ambient Air	N/A: Ambient Air	N/A: Ambient Air
Heart Rate (BPM)	≤59	60–69	70–79	80–129	130–134	135–139	≥140
Systolic BP (mmHg)	≤79	80–84	85–89	90–109	110–119	120–129	≥130
Diastolic BP (mmHg)	≤41	42–47	48–52	53–79	80–85	86–89	≥90
Temperature (°C)	≤34.9	35.3–35.4	35.5–35.9	36–37.0	37.1–37.4	37.5–37.9	≥38
**12–17 Years**							
Respiratory Rate (BPM)	≤9	10–12	13–14	15–24	25–29	30–34	≥35
Saturation (SpO_2_, %)	≤91	92	93	≥94	N/A: Ambient Air	N/A: Ambient Air	N/A: Ambient Air
Heart Rate (BPM)	≤49	50–59	60–69	70–109	110–119	120–129	≥130
Systolic BP (mmHg)	≤89	90–94	95–99	100–119	120–134	135–139	≥140
Diastolic BP (mmHg)	≤41	42–47	48–52	63–80	81–85	86–89	≥90
Temperature (°C)	≤34.9	35.3–35.4	35.5–35.9	36–37.0	37.1–37.4	37.5–37.9	≥38

**Table 3:** **Comparison of interpolation methods showing physiological variables’ maximum range values after interpolation**. We observed a typical distribution of maximum values resulting from polynomial interpolations of orders 3 and 5, noting the occurrence of negative values—anomalous in this context—as well as an implausible order of magnitude. Similar issues were found with cubic spline interpolations. In contrast, linear interpolation displayed a typical distribution in which all values remained positive, with an order of magnitude that appropriately reflected the extreme physiological conditions possible in ICU settings. Therefore, we ultimately selected linear interpolation for our analyses.

	Polynomial (Order 3 and 5) and Cubic Spline Interpolations		Linear Interpolations	
Variable	Minimal	Maximal	Minimal	Maximal
Respiratory rate (breaths/min)	−7.36 × 10^10^	2.51 × 10^14^	0	121
Spo2 (%)	−3.78 × 10^9^	1.81 × 10^14^	0	100
Heart rate (bpm)	−8.62 × 10^13^	2.52 × 10^10^	0	268
Blood pressure systolic (mmhg)	−1.22 × 10^11^	3.82 × 10^10^	0	261
Blood pressure diastolic (mmhg)	−7.30 × 10^10^	5.74 × 10^8^	0	228
MAP(mmhg)	−8.32 × 10^10^	1.07 × 10^10^	0	238

**Table 4:** **Demographics of the study population after preprocessing**. The top panel illustrates age stratification across admissions, revealing no pediatric patients, four teenage admissions, and predominantly adult admissions. Most adults are between the ages of 45–65 or 66–84, with a slightly higher proportion of elderly individuals (66+). Age groups are categorized as infant (0–17 years), adult (18–65 years), and elderly (65+ years). The middle panel shows gender distribution, with a moderate overrepresentation of males compared to females. The bottom panel presents the distribution of race and ethnicity, highlighting an overrepresentation of white individuals. Here, “Hispanic/Latino” is categorized as an ethnicity, while other categories are classified by race.

Category	Subcategory (Years)	Count	Percentage (%)
Age Stratification	<1 year	0	0
	1 to <2	0	0
	2 to 4	0	0
	5 to 11	0	0
	12 to 17	4	0.061369
	18 to 45	836	12.826202
	46 to 65	2369	36.345505
	66 to 85	2693	41.316355
	86+	616	9.450752
Gender	Male	3511	53.866217
	Female	3007	46.133783
Race/Ethnicity	White	4652	71.371586
	Undefined	694	10.647438
	Black/African American	686	10.524701
	Hispanic/Latino	259	3.973612
	Asian	199	3.053084
	American Indian/ Alaska Native	13	0.199448
	Native Hawaiian/ other Pacific Islander	9	0.138079
	Multiracial	6	0.092053

**Table 5:** **Common feature columns** used to train the GBM, RF, and sequential models. Notice that there are also masks for the six different TAGs-score-features. All features are of type float32 (with labels as float64), except for the six TAG features, which are of type int32. The dataset includes a mix of demographic data (e.g., gender, age, ethnicity, and race) and physiological values (e.g., the six main variables such as diastolic and systolic blood pressure, as well as derived physiological features like MAP and BMI). The TAGs-score-features were based on NEWS2+ (refer to Section 2.3.1, labeling and feature engineering), and feature masks were created to indicate values obtained through imputation and interpolation (see Section 2.3.2, handling missing data and masks). Additionally, ethnicity and race features were one-hot encoded. Features with measurement units marked as N/A (Not Applicable) signify that no specific unit exists for the corresponding feature.

Feature Name	Description	Measurement Units
Gender	Biological gender of the patient.	N/A
Age	Age of the patient.	Years
Weight	Body weight of the patient.	Kilograms (kg)
Height	Body height of the patient.	Centimeters (cm)
BMI	Body Mass Index, widely used metric to assess body weight relative to height, providing a quick estimate of body fatness for most individuals.	N/A
*Blood Pressure Systolic*	Systolic blood pressure of the patient.	mmHg
*Blood Pressure Diastolic*	Diastolic blood pressure of the patient.	mmHg
MAP	Mean Arterial Pressure (MAP), an important cardiovascular metric representing the average pressure in a person’s arteries during one cardiac cycle.	mmHg
*Temperature*	Body temperature of the patient.	Degrees Celsius (°C)
*Heart Rate*	Number of heartbeats per minute.	Beats per minute (bpm)
*Respiratory Rate*	Number of breaths per minute.	Breaths per minute
*Oxygen Saturation (SpO* *_2_)*	Oxygen level in the blood.	Percentage (%)
Ethnicity or Race	Ethnicity or race of the patient, including Native Hawaiian/Other Pacific Islander, American Indian/Alaska Native, Black/African American, Asian, Multiracial, Hispanic/Latino (classified as an ethnicity), and White.	N/A
TAG_	Derived feature scores based on NEWS2+ for six key physiological variables: TAG_Heart_Rate, TAG_Temperature, TAG_SpO_2_, TAG_Diastolic_BP, TAG_Systolic_BP, TAG_Respiratory_Rate.	N/A
Mask_	Indicates if values are synthetic (i.e., interpolated or imputed, by 1) or original (by 0) for the following variables: SpO_2_, Systolic_BP, Diastolic_BP, Respiratory Rate, Temperature, BMI, Heart Rate, MAP, Height, Weight, and the six TAG_.	N/A
Label (output to be predicted, as a classification task)	Hypoxemia severity score based on the NEWS2+ scoring matrices for adults without COPD, adults with COPD, and pediatric populations (see part 2.3.1 for the labelization process)	centimeters (cm)

**Table 6:** **Label distribution of hypoxemia severity scores before and after interpolation.** Left columns: before; right columns: after. The count numbers increase due to the minute-by-minute interpolations adding more rows. However, the label distribution, in terms of percentage, remains very similar and retains its characteristics, making it suitable for later training. The imputations (carried out before interpolations) and the interpolations did not change the label distribution.

	Before Interpolation		After Interpolation	
Label (Hypoxemia Severity Score)	Count	Percentage (%)	Count	Percentage (%)
0	729,657	72.2369	17,450,087	76.86217
1	158,419	15.68367	3,293,482	14.50676
2	73,338	7.260548	1,244,804	5.482972
3	48,675	4.818882	714,716	3.1481

**Table 7:** **Default and tuned hyperparameters of the three gradient boosting machine models (GBMs) and Random Forests (RFs).** The hyperparameters were optimized using the HyperOpt library and subsequently added to the existing default parameters. This table presents the search ranges of the hyperparameters used to identify the optimal combination for each model (see tree-based model part Section 3.4.1 for the best results). The default hyperparameters are those used to define our “baseline” models.

Model Classifier (Multi-Class)	Setup (Default Hyperparameters)	Tuned Hyperparameters (HyperOpt Library)
XGBoost	- Objective: ‘multi:softmax’ function - Number of Classes: 4 - Random Seed: 42 - Evaluation Metric: ‘mlogloss’ - Number of Boosting Rounds: 300 - Early Stopping Rounds: 5 - Device: GPU	- max_depth: 4 to 8 - eta: 0.005 to 0.3 - subsample: 0.5 to 1 - gamma: 0 to 22 - min_child_weight: 0 to 15
CatBoost	- Objective: ‘MultiClass’ function - Random Seed: 42 - Iterations: 400 - Evaluation Metric: ‘MultiClass’ - Early Stopping Rounds: 5 - Device: GPU	- depth: 4 to 10 - learning_rate: 0.005 to 0.3 - l2_leaf_reg: 1 to 10 - min_data_in_leaf: 1 to 15
LightGBM	- Objective: ‘multiclass’ function - Number of Classes: 4 - Random Seed: 42 - Number of Boosting Rounds: 300 - Device: GPU	- max_depth: 4 to 8 - learning_rate: 0.005 to 0.3 - bagging_fraction: 0.5 to 1 - min_split_gain: 0 to 22 - min_child_weight: 0 to 15
Random Forest	- Number of Trees in the Forest: 100 - Random State: 42 - Number of Jobs to run in Parallel: −1	- max_depth: 4 to 20 - min_samples_split: 2 to 10 - min_samples_leaf: 1 to 5 - max_features: ‘sqrt’, ‘log2’, or 12 - criterion: ‘gini’ or ‘entropy’

**Table 8:** **Architecture and hyperparameters of the sequential models**. The “masked” layers use an internal Dataloader mask to exclude padded values in sequential input data, ensuring they are not considered during training. Variable-length sequences are handled through packing and unpacking: a mask ignores padding, and *pack_padded_sequence* enables LSTM/GRU layers to process only valid steps. After processing, sequences are unpacked to their original order, preventing padding from affecting learning. Due to long training times, hyperparameters were not extensively optimized; instead, a sufficiently complex architecture was implemented, aimed at fitting the training data well enough to demonstrate proof of concept.

Model Classifier	Architecture and Hyperparameters	Training Configuration
LSTM	- Input Processing: - Input features are masked: x = x × mask - Variable sequence lengths are handled via packing/unpacking sequences - **Recurrent Layers:** - **3-layer LSTM** with: - Input Size: 41 (number of features) - Hidden Size: 256 units per layer - Batch First: True - Fully Connected Layers: - FC1: Linear layer with 256 input and 256 output units - Activation Function: ReLU - FC2: Linear layer with 256 input and 4 output units (number of classes) - **Total Trainable Parameters**: 1,425,668	- Batch Size: 64 - Learning Rate: 0.001 - Number of Epochs: 15 (optimal performance achieved earlier) - Weight Decay: 0.0001 - Training Method: 5-min sliding window with a shift lag of −5 min - Device: GPU (T4 or L4 GPU used)
GRU	- Input Processing: - Input features are masked: x = x × mask - Variable sequence lengths are handled via packing/unpacking sequences - **Recurrent Layers**: - **3-layer GRU** with: - Input Size: 41 (number of features) - Hidden Size: 256 units per layer - Batch First: True - Fully Connected Layers: - FC1: Linear layer with 256 input and 256 output units - Activation Function: ReLU - FC2: Linear layer with 256 input and 4 output units (number of classes) - **Total Trainable Parameters**: 1,085,956	

**Table 9:** **Best fine-tuned models for each GBM and RF following optimization.** This table shows the objective functions under which each model achieved optimal performance on the metrics, along with the best hyperparameter values identified during optimization using HyperOpt-TPE, which define the final models. Area Under ROC Curve (AUC). The names of the hyperparameters are shown as they are. Refer to Table 6 for the range of hyperparameter values explored as the search space for fine-tuning the different models.

Model	Objective Function	Tuned Hyperparameters (Post Fine-Tuning)
Random Forest	AUC	- criterion: entropy - max_depth: 20 - max_features: 12 - min_samples_leaf: 1 - min_samples_split: 7
XGBoost	AUC	- eta: 0.2534 - gamma: 5.1162 - max_depth: 8 - min_child_weight: 4.6473 - subsample: 0.8244
LightGBM	AUC	- bagging_fraction: 0.6408 - learning_rate: 0.2269 - max_depth: 5 - min_child_weight: 4.2377 - min_split_gain: 5.8370
CatBoost	Logloss	- depth: 10 - l2_leaf_reg: 8.1064 - learning_rate: 0.1083 - min_data_in_leaf: 14.9935

**Table 10:** **Comparison of performance metrics for ensemble and sequential models:** Tree-Based Models (GBMs: XGBoost, CatBoost, LightGBM), Random Forests, Voting Classifier ensembles and sequential models (LSTM and GRU) across various configurations. “Soft” and “Hard” refer to the voting methods applied to the Voting Classifier ensembles, which are either composed of the three GBMs or the three GBMs+RF. Notably, all tree-based models use the default parameters from the baseline models (making these ensemble combinations of different tree-based baseline models). Some metrics are presented as averages (avg), where “average” represents the mean result across all four classes for each metric, providing a streamlined overview (for detailed class-specific metrics, see appendix). Consistently high scores underscore the models’ effectiveness in multi-class classification tasks. Optimization of objective functions (AUC and LogLoss) using the HyperOpt library yields slight performance gains, highlighting the impact of hyperparameter tuning. Optimized models are in-dicated with “Opti” as well as the objective function used for this goal. *Note:* Sensitivity is equivalent to recall. No optimization was made for the sequential models.

Model Configuration	Accuracy	Avg Specificity	Macro Avg Precision	Macro Avg Sensitivity (Recall)	Macro Avg F1	Weighted Avg Precision	Weighted Avg Sensitivity (Recall)	Weighted Avg F1	MCC	Avg AUROC	Avg AUPRC
RF Baseline	**0.96**	**0.99**	**0.88**	0.85	0.87	0.96	**0.96**	**0.96**	0.89	**0.995**	0.94
RF Opti AUC	**0.96**	**0.99**	0.85	**0.91**	**0.88**	0.96	**0.96**	**0.96**	0.89	**0.995**	0.94
RF Opti Logloss	**0.96**	0.98	0.85	0.9	0.87	0.96	**0.96**	**0.96**	0.89	**0.995**	0.94
XGBoost Baseline	0.95	0.98	0.85	**0.91**	0.87	0.96	0.95	**0.96**	0.89	**0.995**	0.93
XGBoost Opti AUC	0.95	0.98	0.85	**0.91**	0.87	0.95	0.95	0.95	0.88	**0.995**	0.94
XGBoost Opti LogLoss	0.95	0.98	0.85	**0.91**	0.87	0.96	0.95	**0.96**	0.89	**0.995**	0.94
CatBoost Baseline	**0.96**	0.98	0.85	**0.91**	0.87	0.96	**0.96**	**0.96**	0.89	0.992	0.93
CatBoost Opti AUC	**0.96**	0.98	0.85	**0.91**	0.87	0.96	**0.96**	**0.96**	0.89	**0.995**	0.93
CatBoost Opti LogLoss	0.95	0.98	0.85	**0.91**	0.87	0.96	**0.96**	**0.96**	0.89	**0.995**	0.93
LightGBM Baseline	0.95	0.98	0.85	**0.91**	0.87	0.96	0.95	**0.96**	0.89	**0.995**	0.93
LightGBM Opti AUC	0.95	0.95	0.85	**0.91**	0.87	0.95	0.95	0.95	0.88	**0.995**	0.93
LightGBM Opti LogLoss	0.95	0.98	0.85	**0.91**	0.87	0.96	**0.96**	**0.96**	0.89	**0.995**	0.94
Soft GBMs	**0.96**	**0.99**	**0.88**	0.86	0.87	0.96	**0.96**	**0.96**	0.89	**0.995**	0.94
Soft GBMs + RF	**0.96**	**0.99**	**0.88**	0.86	0.87	0.96	**0.96**	**0.96**	**0.9**	**0.995**	**0.95**
Hard GBMs	**0.96**	0.98	**0.88**	0.86	0.87	0.96	**0.96**	**0.96**	0.89	**0.995**	0.94
Hard GBMs + RF	**0.96**	**0.99**	**0.88**	0.85	0.87	0.96	**0.96**	**0.96**	0.89	**0.995**	0.94
LSTM Model	**0.96**	**0.99**	0.87	0.9	**0.88**	0.96	**0.96**	**0.96**	0.89	**0.995**	0.94
GRU Model	**0.96**	**0.99**	0.87	0.9	**0.88**	**0.97**	**0.96**	**0.96**	**0.9**	**0.995**	**0.95**

**Table 11:** **Comparison of performance metrics for sequential models (LSTM and GRU) across various configurations.** Some metrics are presented as averages, where “average” (or “Avg”) denotes the mean result across all four classes, providing a concise overview (for detailed class-specific metrics, see the Appendix A). Consistently, the high scores highlight these models’ effectiveness in multi-class classification tasks. Due to extensive training times, no fine-tuning was performed; instead, architectures with sufficient complexity were used as a proof of concept in this study. *Note:* Sensitivity is equivalent to recall.

Model Configuration	Accuracy	Avg Specificity	Macro Avg Precision	Macro Avg Sensitivity (Recall)	Macro Avg F1	Weighted Avg Precision	Weighted Avg Sensitivity (Recall)	Weighted Avg F1	MCC	Avg AUROC	Avg AUPRC
LSTM Model	**0.96**	**0.99**	**0.87**	**0.9**	**0.88**	0.96	**0.96**	**0.96**	0.89	**0.995**	0.94
GRU Model	**0.96**	**0.99**	**0.87**	**0.9**	**0.88**	**0.97**	**0.96**	**0.96**	**0.9**	**0.995**	**0.95**

**Table 12:** **Performance comparison of best fine-tuned-tree-based, best voting classifier ensemble and sequential models.** This table compares performance metrics for the best fine-tuned Gradient Boosting Models (XGBoost Opti AUC, CatBoost Opti AUC, and LightGBM Opti LogLoss), RF Opti AUC, soft-GBMS+RF (Voting Classifier Ensemble), and sequential models (LSTM, GRU) across configurations. Indeed, for a fair comparison, GBMs and RF were optimized using HyperOpt TPE with objective functions like AUC and LogLoss, which provided slight performance gains and highlighted the value of hyperparameter tuning in general. Some metrics are shown as averages across all classes, providing a streamlined view (see appendix for detailed class metrics). Both GBMs, RF, and sequential models consistently score high, demonstrating their effectiveness in multi-class classification tasks. Concerning the soft-GBMs+RF ensemble, it showed only slight improvements over-optimized tree-based models. However, it could serve as a viable compromise to sequential models, which will require significantly longer training times in future research. Sequential models also perform well with a 5-min sliding window approach, showing their suitability for time-series classification in general. Due to long training times, sequential models were not fine-tuned but used complex architectures as proof of concept. Note that decimal precision varies in reporting. Both GBMs and sequential models deliver strong, competitive performance in multi-class classification but with pros and cons. See Appendix A Figure A3 for a comparison of all models (including the baseline models).

Model Configuration	Accuracy		Precision Average	Sensitivity Average	Specificity Average	F1-Score Average	Macro Avg Precision	Macro Avg Sensitivity	Macro Avg F1	AUROC Average	AUPRC Average
RF Opti AUC	**0.96**	**0.99**	0.85	**0.91**	**0.88**	0.96	**0.96**	**0.96**	0.89	**0.995**	0.94
XGBoost Opti AUC	0.95	0.98	0.85	**0.91**	0.87	0.95	0.95	0.95	0.88	**0.995**	0.94
CatBoost Opti AUC	**0.96**	0.98	0.85	**0.91**	0.87	0.96	**0.96**	**0.96**	0.89	**0.995**	0.93
LightGBM Opti LogLoss	0.95	0.98	0.85	**0.91**	0.87	0.96	**0.96**	**0.96**	0.89	**0.995**	0.94
Soft GBMs + RF	**0.96**	**0.99**	**0.88**	0.86	0.87	0.96	**0.96**	**0.96**	**0.9**	**0.995**	**0.95**
LSTM Model	**0.96**	**0.99**	0.87	0.9	**0.88**	0.96	**0.96**	**0.96**	0.89	**0.995**	0.94
GRU Model	**0.96**	**0.99**	0.87	0.9	**0.88**	**0.97**	**0.96**	**0.96**	**0.9**	**0.995**	**0.95**

**Figure A2:**
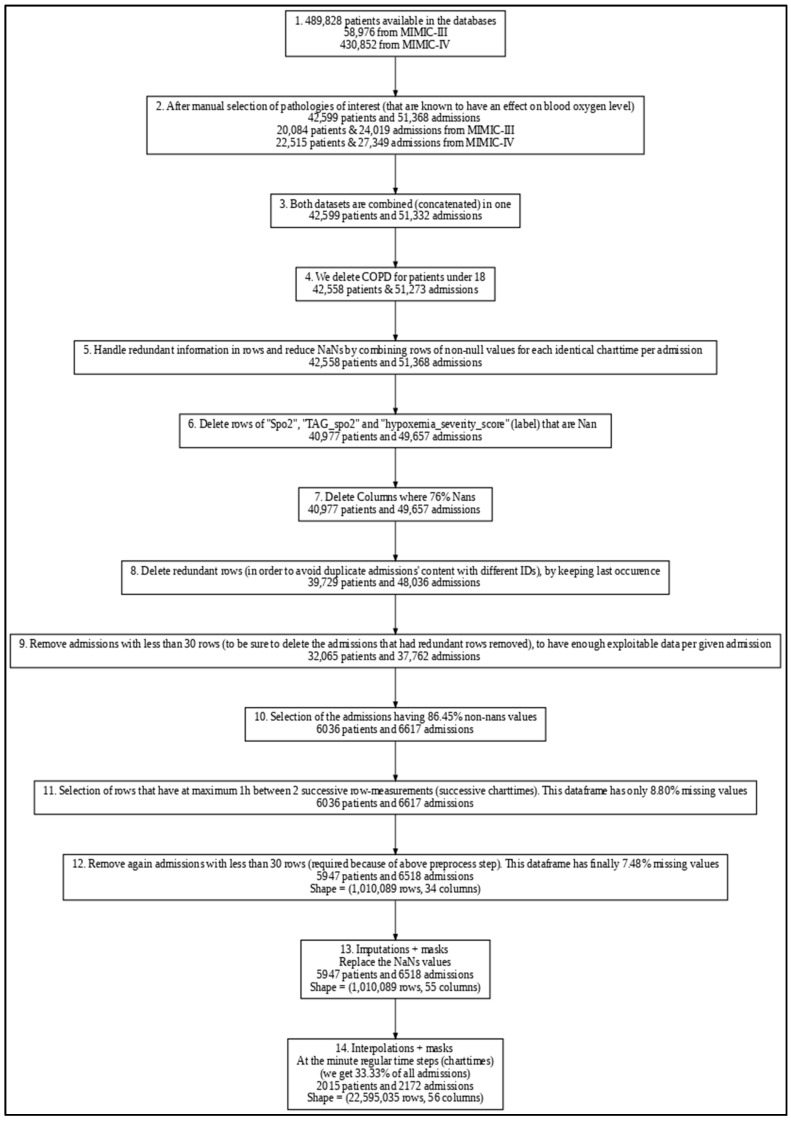
**Inclusion diagram showing the main preprocessing steps before the imputations and interpolations (until point 12) and after (point 13–14).** In summary: (1) Start with raw data from MIMIC-III and MIMIC-IV; (2) Select pathologies of interest from both dataframes to include patients with blood oxygenation issues; (3) Combine both datasets into one large dataframe; (4) Exclude admissions for patients under 18 years old or suffering from chronic obstructive pulmonary disease (COPD) as the proposes EWS, NEWS2+, does not handle these for labeling; (5) Remove redundant rows (artifacts from previous preprocessing steps) by combining rows with non-null values from identical chart time per admission; (6) Delete rows with NaNs in feature columns of interest (SpO_2_ linked) to maximize labeled data; (7) Remove rows where 76% or more values are NaNs; (8) To avoid redundant admissions from combined datasets, for the redundant rows that exist, keep the last occurrence of each concerned row and delete earlier ones; (9) Remove rows with fewer than 30 chart times to ensure sufficient data length. This will also lead to the complete deletion of redundant admissions from the previous step; (10) Select admissions with at least 86.45% non-NaN values; (11) Ensure each admission has a maximum of 1 h between successive rows. Remarkably, this step did not delete any patient or admission from the previous step; (12) Finally, remove admissions with fewer than 30 rows to maintain a certain length; (13) Imputations were applied to replace NaN values with corresponding masks for the affected features, allowing the model to be “informed” about which values are synthetic (i.e., interpolated or imputed) and which are original. This approach ensures the model can distinguish between real and synthetic data during the learning process; (14) Interpolations at the minute level to have regular time steps (in the rows concerned by the charttimes column) with mask for the rows concerned, as well as the features inside it. The resulting dataframe was then used for specific final preprocessing steps tailored for the GBMs and sequential models.

## Data Availability

The data supporting reported results were obtained from the MIMIC-III and MIMIC-IV databases, which are publicly available after an approval procedure at https://mimic.physionet.org/, accessed on 2 December 2024.

## List of Acronyms

SpO_2_	peripheral oxygen saturation.
LSTM	long short-term memory.
PPV	positive predictive value.
SaO2	arterial oxygen saturation.
GBT	gradient boosted tree.
AUROC	area under the receiver operating characteristics.
Lin	linear regression.
LR	logistic regression.
ANN	artificial neural network.
XGB	extreme gradient boosting.
RNN	recurrent neural network.
GBM	gradient boosting model.
PaO_2_	partial pressure of oxygen.
FiO_2_	fraction of oxygen in inhaled gas.
NN	neural network.
RF	random forest.
HR	heart rate.
BR	breath rate.
SBP	systolic blood pressure.
DBP	diastolic blood pressure.
AG	anion gap.
CNN	convolutional neural network.
MAE	mean averaged error to the range of values.
RF	random forest.
TBM	tree-based models.

## Appendix A

The admissions were selected if they included at least one manually chosen pathology of interest from all possible medical conditions, aiming to have a distribution of patients presenting oxygenation issues in the blood. These patients were more likely to experience hypoxemia episodes, thus making their admissions ideal candidates for labeling.

The feature columns used to train the Gradient Boosting Machines (GBMs) and sequential models totaled 41 and 45, respectively. However, for the sequential models, additional input columns—‘charttime’, ‘hadm_id’, and ‘subject_id’—were included. These columns were utilized by the Dataloader to organize the data for sequential processing but were not used directly in training or prediction. Both models used the *hypoxemia_severity_score* column as the target label, and shared a common set of features. This target label was used by the sequential model for the sliding window (5 rows) approach.

## References

[1] Baker D.J. (2005). Critical Care Requirements after Mass Toxic Agent Release. Crit. Care Med..

[2] Carli P., Telion C., Baker D. (2003). Terrorism in France. Prehospital Disaster Med..

[3] Okumura T., Suzuki K., Fukuda A., Kohama A., Takasu N., Ishimatsu S., Hinohara S. (1998). The Tokyo Subway Sarin Attack: Disaster Management, Part 1: Community Emergency Response. Acad. Emerg. Med..

[4] Bourassa S., Paquette-Raynard E., Noebert D., Dauphin M., Akinola P.S., Marseilles J., Jouvet P., Leclerc J. (2022). Gaps in Prehospital Care for Patients Exposed to a Chemical Attack—A Systematic Review. Prehospital Disaster Med..

[5] 39^e^ Congrès de La Recherche Au CHU Sainte-Justine: 5–6 Février 2025. https://recherche.chusj.org/fr/congres2021.

[6] Réseau de Recherche en Santé Respiratoire du Québec. https://rsr-qc.ca/jqrsr-2021/.

[7] Inc M.I.C. Medint Cbrne Group-Groupe Medint Cbrne. https://medintcbrne.com/projects-%26-projets.

[8] Bourassa S. (2023). The Medical Management of Casualties in a Chemical Contaminated Environment: A Start for the CBRNE Defence Research Program for Clinicians. Ph.D. Thesis.

[9] Greenhalgh T., Treadwell J., Ms R.B., Roberts N., Tavare A., Pullyblank A. (2020). Should We Use the NEWS (or NEWS2) Score When Assessing Patients with Possible COVID-19 in Primary Care? Additional Contributors (Topic Experts). https://www.researchgate.net/publication/340934244_Should_we_use_the_NEWS_or_NEWS2_score_when_assessing_patients_with_possible_COVID-19_in_primary_care?channel=doi&linkId=5ea5b751a6fdccd7945721c9&showFulltext=true.

[10] Alam N., Vegting I.L., Houben E., van Berkel B., Vaughan L., Kramer M.H.H., Nanayakkara P.W.B. (2015). Exploring the Performance of the National Early Warning Score (NEWS) in a European Emergency Department. Resuscitation.

[11] Tavaré A., Pullyblank A., Redfern E., Collen A., Barker R.O., Gibson A. (2022). NEWS2 in Out-of-Hospital Settings, the Ambulance and the Emergency Department. Clin. Med..

[12] National Early Warning Score (NEWS) 2. https://www.rcp.ac.uk/improving-care/resources/national-early-warning-score-news-2/.

[13] Paediatric Early Warning Score (PEWS). https://ihub.scot/improvement-programmes/scottish-patient-safety-programme-spsp/maternity-and-children-quality-improvement-collaborative-mcqic/paediatric-care/paediatric-early-warning-score-pews/.

[14] Paediatric Observation Reference Ranges for Referrers. https://www.clinicalguidelines.scot.nhs.uk/rhc-for-health-professionals/referring-a-patient/paediatric-observation-reference-ranges-for-referrers/.

[15] Akre M., Finkelstein M., Erickson M., Liu M., Vanderbilt L., Billman G. (2010). Sensitivity of the Pediatric Early Warning Score to Identify Patient Deterioration. Pediatrics.

[16] Chapman S.M., Maconochie I.K. (2019). Early Warning Scores in Paediatrics: An Overview. Arch. Dis. Child..

[17] Pediatric Vital Signs Normal Ranges|Iowa Head and Neck Protocols. https://medicine.uiowa.edu/iowaprotocols/pediatric-vital-signs-normal-ranges.

[18] Flynn J.T., Kaelber D.C., Baker-Smith C.M., Blowey D., Carroll A.E., Daniels S.R., de Ferranti S.D., Dionne J.M., Falkner B., Flinn S.K. (2017). Clinical Practice Guideline for Screening and Management of High Blood Pressure in Children and Adolescents. Pediatrics.

[19] Validation of a Modified Early Warning Score in Medical Admissions. https://read.qxmd.com/read/11588210/validation-of-a-modified-early-warning-score-in-medical-admissions.

[20] Khan A., Sarma D., Gowda C., Rodrigues G. (2021). The Role of Modified Early Warning Score (MEWS) in the Prognosis of Acute Pancreatitis. Oman Med. J..

[21] Effect of Introducing the Modified Early Warning Score on Clinical Outcomes, Cardio-Pulmonary Arrests and Intensive Care Utilisation in Acute Medical Admissions. https://read.qxmd.com/read/12859475/effect-of-introducing-the-modified-early-warning-score-on-clinical-outcomes-cardio-pulmonary-arrests-and-intensive-care-utilisation-in-acute-medical-admissions.

[22] Smith M.E.B., Chiovaro J.C., O’Neil M., Kansagara D., Quiñones A.R., Freeman M., Motu’apuaka M.L., Slatore C.G. (2014). Early Warning System Scores for Clinical Deterioration in Hospitalized Patients: A Systematic Review. Ann. Am. Thorac. Soc..

[23] Gerry S., Bonnici T., Birks J., Kirtley S., Virdee P.S., Watkinson P.J., Collins G.S. (2020). Early Warning Scores for Detecting Deterioration in Adult Hospital Patients: Systematic Review and Critical Appraisal of Methodology. BMJ.

[24] Downey C.L., Tahir W., Randell R., Brown J.M., Jayne D.G. (2017). Strengths and Limitations of Early Warning Scores: A Systematic Review and Narrative Synthesis. Int. J. Nurs. Stud..

[25] Fu L.-H., Schwartz J., Moy A., Knaplund C., Kang M.-J., Schnock K.O., Garcia J.P., Jia H., Dykes P.C., Cato K. (2020). Development and Validation of Early Warning Score System: A Systematic Literature Review. J. Biomed. Inform..

[26] Shamout F.E., Zhu T., Sharma P., Watkinson P.J., Clifton D.A. (2020). Deep Interpretable Early Warning System for the Detection of Clinical Deterioration. IEEE J. Biomed. Health Inform..

[27] Lauritsen S.M., Kristensen M., Olsen M.V., Larsen M.S., Lauritsen K.M., Jørgensen M.J., Lange J., Thiesson B. (2020). Explainable Artificial Intelligence Model to Predict Acute Critical Illness from Electronic Health Records. Nat. Commun..

[28] Pigat L., Geisler B.P., Sheikhalishahi S., Sander J., Kaspar M., Schmutz M., Rohr S.O., Wild C.M., Goss S., Zaghdoudi S. (2024). Predicting Hypoxia Using Machine Learning: Systematic Review. JMIR Med. Inform..

[29] Johnson A., Pollard T., Mark R. MIMIC-III Clinical Database 2015. https://physionet.org/content/mimiciii/1.4/.

[30] Johnson A.E.W., Pollard T.J., Shen L., Lehman L.H., Feng M., Ghassemi M., Moody B., Szolovits P., Anthony Celi L., Mark R.G. (2016). MIMIC-III, a Freely Accessible Critical Care Database. Sci. Data.

[31] Johnson A.E.W., Bulgarelli L., Shen L., Gayles A., Shammout A., Horng S., Pollard T.J., Hao S., Moody B., Gow B. (2023). MIMIC-IV, a Freely Accessible Electronic Health Record Dataset. Sci. Data.

[32] Ke G., Meng Q., Finley T., Wang T., Chen W., Ma W., Ye Q., Liu T.-Y. (2017). LightGBM: A Highly Efficient Gradient Boosting Decision Tree. Proceedings of the Advances in Neural Information Processing Systems.

[33] Prokhorenkova L., Gusev G., Vorobev A., Dorogush A.V., Gulin A. (2018). CatBoost: Unbiased Boosting with Categorical Features. Adv. Neural Inf. Process. Syst..

[34] Chen T., Guestrin C. (2016). XGBoost: A Scalable Tree Boosting System. Proceedings of the 22nd ACM SIGKDD International Conference on Knowledge Discovery and Data Mining.

[35] Gers F.A., Schmidhuber J., Cummins F. Learning to Forget: Continual Prediction with LSTM. Proceedings of the 1999 Ninth International Conference on Artificial Neural Networks ICANN 99, (Conf. Publ. No. 470).

[36] Chung J., Gulcehre C., Cho K., Bengio Y. Empirical Evaluation of Gated Recurrent Neural Networks on Sequence Modeling. https://arxiv.org/abs/1412.3555v1.

[37] Hochreiter S., Schmidhuber J. (1997). Long Short-Term Memory. Neural Comput..

[38] Dempsey J.A., Wagner P.D. (1999). Exercise-Induced Arterial Hypoxemia. J. Appl. Physiol..

[39] Johannigman J., Gerlach T., Cox D., Juhasz J., Britton T., Elterman J., Rodriquez D., Blakeman T., Branson R. (2015). Hypoxemia during Aeromedical Evacuation of the Walking Wounded. J. Trauma Acute Care Surg..

[40] Bourassa S., Bouchard P.-A., Dauphin M., Lellouche F. (2020). Oxygen Conservation Methods with Automated Titration. Respir. Care.

[41] Samad M.D., Abrar S., Diawara N. (2022). Missing Value Estimation Using Clustering and Deep Learning within Multiple Imputation Framework. Knowl.-Based Syst..

[42] Perez-Lebel A., Varoquaux G., Le Morvan M., Josse J., Poline J.-B. (2022). Benchmarking Missing-Values Approaches for Predictive Models on Health Databases. GigaScience.

[43] Josse J., Chen J.M., Prost N., Scornet E., Varoquaux G. (2024). On the Consistency of Supervised Learning with Missing Values. Stat. Pap..

[44] Sharafoddini A., Dubin J.A., Maslove D.M., Lee J. (2019). A New Insight Into Missing Data in Intensive Care Unit Patient Profiles: Observational Study. JMIR Med. Inform..

[45] Sperrin M., Martin G.P., Sisk R., Peek N. (2020). Missing Data Should Be Handled Differently for Prediction than for Description or Causal Explanation. J. Clin. Epidemiol..

[46] Kusters R., Misevic D., Berry H., Cully A., Le Cunff Y., Dandoy L., Díaz-Rodríguez N., Ficher M., Grizou J., Othmani A. (2020). Interdisciplinary Research in Artificial Intelligence: Challenges and Opportunities. Front. Big Data.

[47] Lundberg S.M., Nair B., Vavilala M.S., Horibe M., Eisses M.J., Adams T., Liston D.E., Low D.K.-W., Newman S.-F., Kim J. (2018). Explainable Machine-Learning Predictions for the Prevention of Hypoxaemia during Surgery. Nat. Biomed. Eng..

[48] Annapragada A.V., Greenstein J.L., Bose S.N., Winters B.D., Sarma S.V., Winslow R.L. (2021). SWIFT: A Deep Learning Approach to Prediction of Hypoxemic Events in Critically-Ill Patients Using SpO2 Waveform Prediction. PLOS Comput. Biol..

[49] Bureau U.C. About the Topic of Race. https://www.census.gov/topics/population/race/about.html.

[50] Lassman D., Hartman M., Washington B., Andrews K., Catlin A. (2014). US Health Spending Trends By Age And Gender: Selected Years 2002–2010. Health Aff..

[51] González-Nóvoa J.A., Busto L., Rodríguez-Andina J.J., Fariña J., Segura M., Gómez V., Vila D., Veiga C. (2021). Using Explainable Machine Learning to Improve Intensive Care Unit Alarm Systems. Sensors.

[52] Breiman L. (2001). Random Forests. Mach. Learn..

[53] Hyland S.L., Faltys M., Hüser M., Lyu X., Gumbsch T., Esteban C., Bock C., Horn M., Moor M., Rieck B. (2020). Early Prediction of Circulatory Failure in the Intensive Care Unit Using Machine Learning. Nat. Med..

[54] Zheng J., Li J., Zhang Z., Yu Y., Tan J., Liu Y., Gong J., Wang T., Wu X., Guo Z. (2023). Clinical Data Based XGBoost Algorithm for Infection Risk Prediction of Patients with Decompensated Cirrhosis: A 10-Year (2012–2021) Multicenter Retrospective Case-Control Study. BMC Gastroenterol..

[55] Zhao H., Ma Z., Sun Y. Predict Onset Age of Hypertension Using CatBoost and Medical Big Data. Proceedings of the 2020 International Conference on Networking and Network Applications (NaNA).

[56] Pham T.D. (2021). Time–Frequency Time–Space LSTM for Robust Classification of Physiological Signals. Sci. Rep..

[57] Lipton Z.C., Kale D.C., Elkan C., Wetzel R. (2015). Learning to Diagnose with LSTM Recurrent Neural Networks. arXiv.

[58] Mishra S., Tiwari N.K., Kumari K., Kumawat V. Prediction of Heart Disease Using Machine Learning. Proceedings of the 2023 2nd International Conference on Applied Artificial Intelligence and Computing (ICAAIC).

[59] Moreno-Sanchez P.A. Development of an Explainable Prediction Model of Heart Failure Survival by Using Ensemble Trees. Proceedings of the 2020 IEEE International Conference on Big Data (Big Data).

[60] Temel G., Ankarali H., Taşdelen B., Erdoğan S., Özge A. (2014). A Comparison of Boosting Tree and Gradient Treeboost Methods for Carpal Tunnel Syndrome. Turk. Klin. J. Biostat..

[61] Yang Y. Prediction of Blood Oxygen Saturation Based on Deep Learning. Proceedings of the International Conference on Algorithms, Microchips and Network Applications.

[62] Ma F., Chitta R., Zhou J., You Q., Sun T., Gao J. Dipole: Diagnosis Prediction in Healthcare via Attention-Based Bidirectional Recurrent Neural Networks. Proceedings of the 23rd ACM SIGKDD International Conference on Knowledge Discovery and Data Mining.

[63] Parkinson’s Disease Detection Using Hybrid LSTM-GRU Deep Learning Model. https://www.mdpi.com/2079-9292/12/13/2856.

[64] Suo Q., Ma F., Canino G., Gao J., Zhang A., Veltri P., Agostino G. (2018). A Multi-Task Framework for Monitoring Health Conditions via Attention-Based Recurrent Neural Networks. AMIA. Annu. Symp. Proc..

[65] Shwartz-Ziv R., Armon A. (2022). Tabular Data: Deep Learning Is Not All You Need. Inf. Fusion.

[66] Weitz J.I., Fredenburgh J.C., Eikelboom J.W. (2017). A Test in Context: D-Dimer. J. Am. Coll. Cardiol..

[67] Bouillon-Minois J.-B., Roux V., Jabaudon M., Flannery M., Duchenne J., Dumesnil M., Paillard-Turenne M., Gendre P.-H., Grapin K., Rieu B. (2021). Impact of Air Transport on SpO2/FiO2 among Critical COVID-19 Patients during the First Pandemic Wave in France. J. Clin. Med..

[68] Chen W., Janz D.R., Shaver C.M., Bernard G.R., Bastarache J.A., Ware L.B. (2015). Clinical Characteristics and Outcomes Are Similar in ARDS Diagnosed by Oxygen Saturation/Fio2 Ratio Compared with Pao2/Fio2 Ratio. Chest.

[69] Faltys M., Zimmermann M., Lyu X., Hüser M., Hyland S., Rätsch G., Merz T. HiRID, a High Time-Resolution ICU Dataset. https://physionet.org/content/hirid/1.1.1/.

